# The Hyperthermophilic Restriction-Modification Systems of *Thermococcus kodakarensis* Protect Genome Integrity

**DOI:** 10.3389/fmicb.2021.657356

**Published:** 2021-05-20

**Authors:** Kelly M. Zatopek, Brett W. Burkhart, Richard D. Morgan, Alexandra M. Gehring, Kristin A. Scott, Thomas J. Santangelo, Andrew F. Gardner

**Affiliations:** ^1^New England Biolabs, Ipswich, MA, United States; ^2^Department of Biochemistry and Molecular Biology, Colorado State University, Fort Collins, CO, United States

**Keywords:** *Archaea*, restriction-modification system, genome integrity, viral defense mechanisms, methyltransferase

## Abstract

*Thermococcus kodakarensis* (*T. kodakarensis*), a hyperthermophilic, genetically accessible model archaeon, encodes two putative restriction modification (R-M) defense systems, TkoI and TkoII. TkoI is encoded by TK1460 while TkoII is encoded by TK1158. Bioinformative analysis suggests both R-M enzymes are large, fused methyltransferase (MTase)-endonuclease polypeptides that contain both restriction endonuclease (REase) activity to degrade foreign invading DNA and MTase activity to methylate host genomic DNA at specific recognition sites. In this work, we demonsrate *T. kodakarensis* strains deleted for either or both R-M enzymes grow more slowly but display significantly increased competency compared to strains with intact R-M systems, suggesting that both TkoI and TkoII assist in maintenance of genomic integrity *in vivo* and likely protect against viral- or plasmid-based DNA transfers. Pacific Biosciences single molecule real-time (SMRT) sequencing of *T. kodakarensis* strains containing both, one or neither R-M systems permitted assignment of the recognition sites for TkoI and TkoII and demonstrated that both R-M enzymes are TypeIIL; TkoI and TkoII methylate the N^6^ position of adenine on one strand of the recognition sequences GTGAAG and TTCAAG, respectively. Further *in vitro* biochemical characterization of the REase activities reveal TkoI and TkoII cleave the DNA backbone GTGAAG(N)_20_/(N)_18_ and TTCAAG(N)_10_/(N)_8_, respectively, away from the recognition sequences, while *in vitro* characterization of the MTase activities reveal transfer of tritiated S-adenosyl methionine by TkoI and TkoII to their respective recognition sites. Together these results demonstrate TkoI and TkoII restriction systems are important for protecting *T. kodakarensis* genome integrity from invading foreign DNA.

## Introduction

Across all domains of life, organisms have evolved a variety of innate defense systems that provide protection against invading foreign DNA. One of the most well characterized defense systems is the restriction–modification (R-M) system found in prokaryotic organisms (Loenen et al., 2014). R-M systems protect against invading DNA, such as viral DNA, and are comprised of two components, a restriction endonuclease (REase) and a methyltransferase (MTase). The REase component has Mg^2+^ dependent endonucleolytic activity that cleaves the phosphodiester backbone of DNA at a defined site, initiating degradation. The MTase component transfers a methyl group from a co-factor, typically S-adenosyl methionine (SAM), to host genomic DNA at a defined recognition site to allow host to distinguish self from invading DNA. Methylation is typically at either the C^5^ or N^4^ position of cytosine or N^6^ position of adenine. The action of these two enzymatic activities together both protects host genomic DNA and initiates degradation of invading foreign DNA. R-M systems are classified into four main groups, Type I–Type IV, based on organization and co-factor requirements, and each class has different cleavage-methylation patterns. For example, in Type I R-M systems the REase cleaves DNA at random sites far from the MTase recognition site, while in Type II R-M systems the REase cleaves DNA just outside the MTase recognition site.

The breadth and distribution of R-M systems has been widely expanded in the last decade due to the emergence of next generation sequencing (NGS) technologies that allow for the detection of methylated bases during whole genome sequencing. Pacific Bioscience (PacBio) single-molecule real-time (SMRT) sequencing allows for the direct detection of the most common methylated bases resulting from MTase activity, N^4^-cytosine (4mC) and N^6^-adenine (6mA). During SMRT-sequencing, 6mA and 4mC can be reliably detected as they cause a pause in the PacBio sequence polymerase interpulse duration (IPD) (Flusberg et al., 2010). The IPD is then utilized to predict putative 4mC and 6mA methylation motifs. Genome-wide mapping of methylation motifs via SMRT sequencing thus provides an immediate and defining signal to identify new MTase methylation motifs, allowing for the discovery of unknown methylases and R-M systems (Clark et al., 2012; Murray et al., 2012; Blow et al., 2016; Zatopek et al., 2018). As a result, PacBio SMRT sequencing and modification detection is currently the preferred first step in identifying new restriction-modification systems and indicates the presence of active systems worth investigating. Methylated motifs can be then matched to their particular MTase genes through cloning or mutation analysis.

Single-molecule real-time sequencing, homology-based database searching, and the development of REBASE, a database dedicated to R-M systems (Roberts et al., 2015), has expanded our understanding of the diversity and distribution of R-M systems in Bacteria, and increasingly, in Archaea. New tools have discovered R-M systems across diverse archaeal clades, including *Sulfolobus acidocaldarius* and *Haloferax volcanii* (Couturier and Lindas, 2018; Ouellette et al., 2018). Currently, 2609 R-M systems have been predicted from 309 archaeal organisms, although only a handful of these archaeal R-M systems have been characterized; for an updated list please visit: http://rebase.neb.com/rebase/arcbaclistA.html (Nolling and De Vos, 1992; Pingoud et al., 2003).

*Thermococcus kodakarensis* is a hyperthermophilic, marine archaeon with a growth temperature range from 60 to 100°C and optimal growth at 85°C (Atomi et al., 2004). Previous studies on the uptake of plasmid DNA by *T. kodakarensis* have noted that the transformation efficiency of unmodified plasmid DNA is lower than methylated plasmids, suggesting the presence of innate defense systems against foreign DNA (Fukui et al., 2005). Indeed, the *T. kodakarensis* genome encodes a variety of putative cellular defense systems including genes annotated as components of the abortive infection/phage exclusion systems (ABI), Bacteriophage Exclusion (BREX), Clustered regularly interspaced short palindromic repeats with cas genes (CRISPR-CAS), R-M systems, Defense island system associated with restriction–modification (DISARM), DNA phosphorothioation (DND), and Toxin-Antitoxin, amongst others (Makarova et al., 2013; Zhang et al., 2020) (Supplementary Table 1). The retention of such a diverse range of host defense systems adumbrates their biological value, but to date, the importance and activities of few, if any of these systems have been evaluated with combined genetic and biochemical investigations.

In this work we assign function and biological importance to the two R-M systems in *T. kodakarensis*, encoded by TK1460 and TK1158, and annotated as TkoI and TkoII in REBASE, respectively. Through sequencing, cloning, expression and biochemical characterization of TkoI and TkoII, we demonstrate that both are members of a unique class of Type II R-M systems, denoted Type IIL (Pingoud et al., 2014). These Type IIL R-M enzymes are fusion endonuclease-MTases, with both domains in a single polypeptide chain, and the MTase methylates a single, or lone strand of the recognition sequence. SMRT sequencing and *in vitro* MTase characterization reveal TkoI methylates GTGAAG and TkoII methylates TTCAAG, both at the N^6^ position of adenine, while *in vitro* characterization of the REase activity shows dsDNA cleavage occurs distant from the MTase recognition site. *T. kodakarensis* strains lacking TkoI, TkoII, or both R-M enzymes show slower growth at 95°C, suggesting an important role for these R-M enzymes for cellular fitness. In addition, *T. kodakarensis* strains lacking TkoI and TkoII have increased transformation efficiency of unmodified foreign plasmid DNA. Taken together, these results suggest a critical role for R-M systems in protecting and maintaining the archaeal *T. kodakarensis* genome, and further highlight the diversity and span of R-M systems across life.

## Materials and Methods

### *Thermococcus kodakarensis* Strain Construction, Growth, and Transformation

Standard procedures were used to markerlessly delete TkoI (encoded by TK1460) and TkoII (encoded by TK1158) from the genome of *T. kodakarensis* strain TS559 (Hileman and Santangelo, 2012; Gehring et al., 2017). Briefly, a non-replicating plasmid was transformed into TS559 and was integrated into the genome at the target loci (TK1460 or TK1158) via recombination; transformants were identified by co-integration of a selectable marker restoring agmatine prototrophy. The intermediate strains containing the integrated plasmid were confirmed using diagnostic PCR on purified genomic DNA. The intermediate cells were then grown in the presence of agmatine as well as 6-methylpurine (the counter-selectable marker), and strains were identified wherein the plasmid had excised from the genomic loci. Again, diagnostic PCR using locus-specific primers on purified genomic DNA from these final strains was used to determine if the target locus was deleted from the genome or if the parent genome (TS559) had been restored. The strain lacking both TkoI and TkoII was constructed following the same process, but consecutively, first deleting TkoII and then deleting TkoI. Whole genome sequencing (see below) using the PacBio Sequel Sequencing platform (Pacific Biosciences, Menlo Park, CA, United States) confirmed the deletion of TK1460 and TK1158 (Supplementary Figure 1).

All *T. kodakarensis* strains were grown anaerobically in artificial seawater supplemented with 5 g/L yeast extract, 5 g/L tryptone, and 2 g/L of sulfur (Fukui et al., 2005; Santangelo et al., 2007; Gehring et al., 2017). Culture growth was monitored by optical density at 600 nm. Growth on Gelrite-solidified medium, formulated as previously described (Santangelo et al., 2007; Gehring et al., 2017), resulted in visible colonies after 48 to 96 h of incubation under anaerobic conditions at 85°C.

Transformation procedures resulting in genomic modifications or retention of autonomously replicated vectors were performed as described (Gehring et al., 2017). Conversion of tryptophan prototrophic recipient cells to tryptophan auxotrophic strains through transformation and retention of the autonomously replicating plasmid pLC71 (Santangelo et al., 2008) was used for plasmid-based transformations. pLC71 contains a combined total of nine recognition motifs for TkoI and TkoII; four for TkoI and five for TkoII.

### Pacific Biosciences Library Construction and SMRT Sequencing

Genomic DNA was purified from *T. kodakarensis* strains TS559, ΔTkoI, ΔTkoII, and ΔTkoI/ΔTkoII using a Monarch Genomic DNA purification Kit (New England Biolabs, Ipswich, MA, United States). 1 μg of genomic DNA from each strain was sheered into 10 kb fragments using a Megaruptor 2 DNA fragmentation instrument (Diagenode Denville, NJ, United States). Following a size selection using AMPure PB beads (Pacific Biosciences) standard 10 kb PacBio library construction was performed utilizing NEB reagents. Briefly, sheered genomic DNA, eluted in 42 μL of TE light buffer (10 mM Tris–HCl, pH 7.5, 0.1 mM EDTA), was incubated with Endonuclease VII (10 units) for 20 min at 37°C in NEBNext FFPE DNA Repair Buffer, followed by the addition of 2 μL FFPE DNA repair mix for 30 min at 37°C, and 5 μL of NEBNext End Repair Mix for 5 min at room temperature. Following an AMPure PB bead clean-up and elution of repaired genomic DNA into 42 μL of TE light, blunt adapters (2.5 μL of 80 μM stock) were ligated onto DNA for 1 hr at RT in 1× T4 DNA ligation buffer using T4 DNA ligase (2000 units). After heat denaturation of T4 DNA ligase, DNA libraries were incubated with Exonuclease III (100 units) and Exonuclease VII (10 units) for 1 h at 37°C. Exonuclease III and Exonuclease VII degrade linear single- and double-stranded DNA that is incompletely ligated to PacBio hairpin adaptors. Three AMPure bead clean-ups were performed and DNA libraries were eluted in 20 μL TE light buffer and quantified using a Qubit fluorometer. TS559 and ΔTkoI libraries were sequenced on an RSII instrument using Magbead loading and P6 Polymerase chemistry for 360 min; ΔTkoII and ΔTkoI/ΔTkoII libraries were barcoded during ligation, pooled and run on a Sequel instrument in a single-cell using diffusion loading and Polymerase 2.0 chemistry for 600 min. Secondary PacBio Resequencing Analysis of deletion strains (ΔTkoI, ΔTkoII, and ΔTkoI/ΔTkoII) to the parental TS559 strain was performed to confirm deletion of each gene (Supplementary Figure 1). Secondary PacBio Modification and Motif analysis was performed to identify 6mA methylation motifs. During PacBio SMRT sequencing, the instrument records the IPD for every base sequenced. Importantly, methylated bases (6mA and 4mC) cause a pause in the IPD. Secondary Modification and Motif analysis calculates the IPD ratio (Experimental IPD/Expected IPD) for every base sequenced and utilizes the observed increase in IPD ratio at methylated bases to predict methylation motifs (Flusberg et al., 2010).

### TkoI and TkoII Cloning, Expression and Purification

An *Escherichia coli* codon optimized version of the genes encoding *T. kodakarensis* TkoI and TkoII were synthetically constructed and cloned into a pET29a vector via *Nde*I and *Bam*HI sites (Genscript Piscataway, NJ, United States). Sequence-confirmed plasmids containing the TkoI or TkoII gene sequences were transformed into NiCo21(DE3) competent cells (New England Biolabs), grown in 1 L of LB + kanamycin (80 μg/mL final) at 37°C to an OD600 of ∼0.4–0.6 and protein-expression was induced with 0.4 mM (final) IPTG. Following growth for an additional 3 h at 37°C, cells were harvested by centrifugation at 4,500 × *g* for 20 min. Cell pellets were resuspended in 150 mL Buffer A (20 mM Tris–HCl pH 7.5, 300 mM NaCl) and lysed using a Constant cell disruptor (Constant Systems Ltd, Northants, United Kingdom). Lysates were heated to 80°C for 20 min and cell debris was removed by centrifugation at 35,000 × *g* for 20 min.

For each protein, clarified supernatant was loaded onto a 16/10 HiPrep DEAE column (GE Life Sciences, Pittsburg, PA, United States), the flow-through was collected and loaded onto a 5 mL HisTrap FF column (GE Life Sciences) and bound proteins were eluted with a 60 mL linear gradient to Buffer B (20 mM Tris–HCl pH 7.5, 300 mM NaCl, 0.5 M imidazole). TkoI or TkoII containing fractions were identified by SDS page, pooled and dialyzed into storage buffer (100 mM KCl, 10 mM Tris–HCl, 1 mM DTT, 0.1 mM EDTA, and 50% glycerol at pH 7.4).

### TkoI and TkoII *in vitro* MTase Activity Assays

To confirm the *in vitro* MTase activity of TkoI and TkoII, a ^3^H-S-adenosyl-methionine MTase assay was performed. A 206-bp TK1460 substrate (TkoI oligo) was generated via PCR from *T. kodakarensis* genomic DNA with primers 1460_substrate_F (5′-TAT CGG GAA TGC GTT CCT CAT AAG GAT GAC G) and 1460_substrate_R (5′-CGA TAT TCA CAG TTG ATG ACC TCG CCA GGG CTC); a 206-bp TK1158 substrate (TkoII oligo) was generated via PCR from *T. kodakarensis* genomic DNA with primers 1158_substrate_F (5′-CGT GTA GGA ACT GGT AGA TTG AGT AGG CGC TTG) and 1158_substrate_R (5′-AGT AGA GGA AGA CGA AAT TAG AAT CTC AGA AG). TkoI oligo contains a single, centrally located recognition sequence for TkoI (GTGAAG) but no recognition sequence for TkoII, while TkoII oligo contains a single, centrally located recognition sequence for TkoII (TTCAAG) but no recognition sequence for TkoI. 1 μg of either amplicon substrate (∼7.5 pmoles) was combined with 1 μM TkoI (or TkoII) and 1 μM S-adenosyl-methionine (or 1 μM ^3^H-S-adenosyl-methionine) (PerkinElmer, NET155V250UC) in a 30 μl reaction containing 50 mM Tris–HCl pH 7.4, 5 mM MgCl_2_, 1 mM β-mercaptoethanol, 50 mM NaCl at 85°C for 2 h. Reactions were terminated by addition of 170 μl H_2_O and 70 μl of a 25:24:1 phenol/chloroform/isoamyl (PCI) alcohol mixture and vigorous mixing. Following separation of the aqueous phase and a second 70 μl PCI extraction, the aqueous phase was filtered through a Nanosep 10 K Omega filtration column (OD010C34). Bound substrates were centrifugally washed five times with 300 μl H_2_O per wash before recovery from the filter and scintillation counting to quantify transfer of ^3^H-methyl groups to the DNA substrates.

### TkoI and TkoII *in vitro* Endonuclease Activity Assay

Previous REase cleavage assays using Type IIL R-M enzymes showed the REase activity of this class requires SAM as well as Supplementary trans-DNA for optimal REase cleavage (Morgan et al., 2008, 2009). Therefore, all *in vitro* REase cleavage reactions were performed in the presence of SAM and trans-DNA. 90mer hairpin oligonucleotides containing the R-M recognition motif (underlined) with the sequence 5′ TTG ATC ACG GTA ACC GAT CAG GTG AAG AAC AAG CCC GAA TTC ACC CTT TTT GGG TGA ATT CGG GCT TGT TCT TCA CCT GAT CGG TTA CCG TGA TCAA-3′ for TkoI and 5′-TTG ATC ACG GTA ACC GAT CAG TTC AAG AAC AAG CCC GAA TTC ACC CTT TTT GGG TGA ATT CGG GCT TGT TCT TGA ACT GAT CGG TTA CCG TGA TCAA-3′ for TkoII were ordered from Integrated DNA Technologies (Coralville, IA, United States) and served as trans-DNA for REase activity reactions. Trans-DNA was annealed in 1X annealing buffer (20 mM Tris–HCl, 100 mM NaCl, pH 7.5) to a final concentration of 5 μM by heating to 95°C for 3 min following by cooling to room temperature.

TkoI and TkoII REase activity was assayed by mixing TkoI (400 nM) or TkoII (10 nM) with plasmid pBR322 (1 μg) or pUC19 (3 μg), SAM (80 μM), 90mer trans-DNA (0.2 μM) in 1× NEBuffer 3 (100 mM NaCl, 50 mM Tris–HCl, pH 7.9, 10 mM MgCl_2_) for TkoI or NEBuffer 1 (10 mM Bis-Tris–Propane-HCl, pH 7, 10 mM MgCl_2_, 1 mM DTT) for TkoII in a 50 μL reaction and incubated at 65°C for 30 min. For pBR322 reactions, four two-fold serial dilutions were performed. For both pBR322 and pUC19 reactions, a negative control was performed in which the R-M enzyme was excluded from the reaction. Reactions were halted by adding 0.5 units of Proteinase K and incubating for 30 min at 37°C followed by the addition of 10 μL of 6× Purple Loading dye and separation on a 1% agarose gel. To identify the REase cut site, the linear product band was excised from the pUC19 digestion gel, DNA was purified using the Monarch DNA Gel Extraction Kit and analyzed by Sanger Sequencing.

## Results

### Bioinformatic Identification of TkoI and TkoII

**FIGURE 1:**
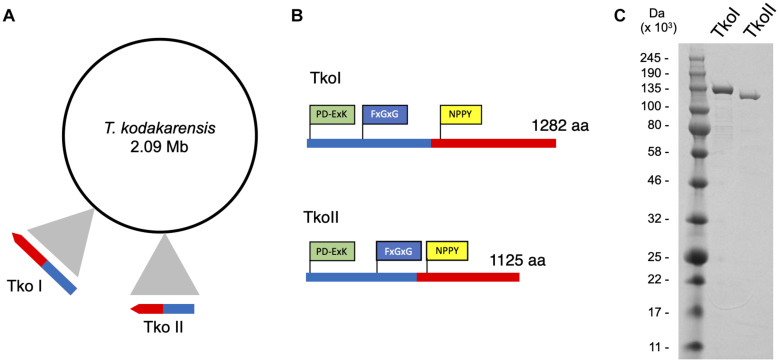
Genomic organization of *Thermococcus kodakarensis* restriction-modification systems. **(A)** ORF TK1460 (1,285,915–1,289,763 nt) encodes TkoI and ORF TK1158 (1,017,279–1,020,656 nt) encodes TkoII endonuclease-methyltransferase fusion proteins. **(B)** TkoI (1,282 aa) and TkoII (1,125 aa) contain a conserved endonuclease motif PD-ExK (green) and FxGxG and NPPY methyltransferase motifs (blue and yellow, respectively). **(C)** SDS-PAGE of purified TkoI and TkoII demonstrate the recombinant proteins are thermo-tolerant, easily purified and >95% pure.

REBASE, an open source on-line database of DNA MTases, R-M systems and associated proteins identified two putative DNA MTases in the *T. kodakarensis* genome encoded by genes TK1460 and TK1158 (TkoI and TkoII, respectively) (Figure 1). Moreover, REBASE categorized these genes as fusion DNA MTases and DNA endonucleases, suggesting both TkoI and TkoII are putative Type IIG R-M systems having both MTase and endonuclease activities (Pingoud et al., 2014). TkoI and TkoII contain an N-terminal endonuclease PD-ExK motif and more centrally located methylase motifs including a signature SAM binding motif (NPPY) and a catalytic FxGxG motif (Figure 1 and Supplementary Figures 2, 3). Despite these bioinformatic predictions, the activities and recognition sites of these enzymes have not been characterized.

### Characterization of TkoI and TkoII Methyltransferase Recognition Sites and Activities

**FIGURE 2:**
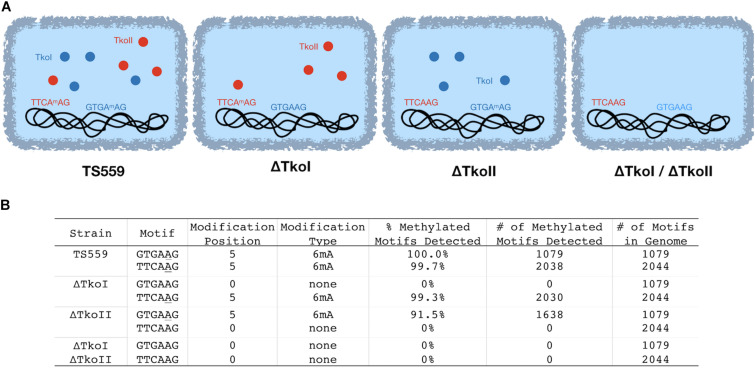
TkoI and TkoII methylate single strands of defined DNA motifs *in vivo*. PacBio SMRT sequencing was used to sequence genomic DNA from four *T. kodakarensis* strains (TS559, ΔTkoI, ΔTkoII, and ΔTkoI/ΔTkoII). **(A)** *T. kodakarensis* strains are schematically illustrated with TkoI (blue) and TkoII (red) and detected modification motifs in each strain are highlighted. **(B)** After sequencing, PacBio Modification and Motif software identified sequence motifs that were modified in each strain.

In the past, new restriction-modification systems were discovered by cloning, expressing and characterizing REases and cognate MTases and then mapping cleavage activity on known substrates to identify the recognition site (Loenen et al., 2014). More recently, PacBio SMRT sequencing has emerged as a gold standard for the rapid discovery of new restriction and modification systems due to the ability to quickly identify methylated motifs in a genome sequence that result from host modification MTases. For example, Blow et al. (2016) identified over two hundred putative restriction modification sites by sequencing genomic DNA with PacBio SMRT sequencing and identifying methylated motifs. To identify candidate methylation motifs in the *T. kodakarensis* genome, we used PacBio SMRT-sequencing to directly detect DNA modifications such as 6mA and 4mC methylation using standard PacBio Modification and Motif analysis software. In the parental strain TS559, encoding both TkoI and TkoII, we identified two methylation motifs by PacBio sequencing (GTGA**A**G and TTCA**A**G, wherein the bold A denotes the location of 6mA) (Figure 2 and Supplementary Figure 4A). Across the *T. kodakarensis* genome, 6mA was identified in 2038 out of 2044 TTCAAG sites and in all (1079/1079) GTGAAG sites. The 2 Mbp *T. kodakarensis* genome would only be predicted to contain ∼500 of each motif (46 = 4096) by random chance and the retention of ∼4 times this number of motifs suggests a biological role in defining host- versus invader-DNA. Interestingly, for both motifs only one strand of the motif was methylated, suggesting both R-M systems are a subtype of Type IIG, classified as Type IIL systems for “lone” strand modification (Supplementary Figure 4A) (Morgan et al., 2008, 2009). Despite very clear site-specific and motif-restricted methylations across the entire genome, the enzymes that modify each motif were unknown. To assign TkoI and TkoII to a specific modification motif, we repeated the PacBio sequencing with genomic DNA from *T. kodakarensis* strains that lacked either TkoI or TkoII. Deletion of TkoI (TK1460) abolished 6mA methylation at GTGAAG sites, but methylation at TTCAAG motifs were unaffected. Deletion of TkoII (TK1158) abolished 6mA methylation at TTCAAG sites but did not impact methylation at GTGAAG motifs (Figure 2 and Supplementary Figures 4B,C). As expected, strains that lacked both TkoI and TkoII abolished methylation at both motifs (Figure 2 and Supplementary Figure 4D).

**FIGURE 3:**
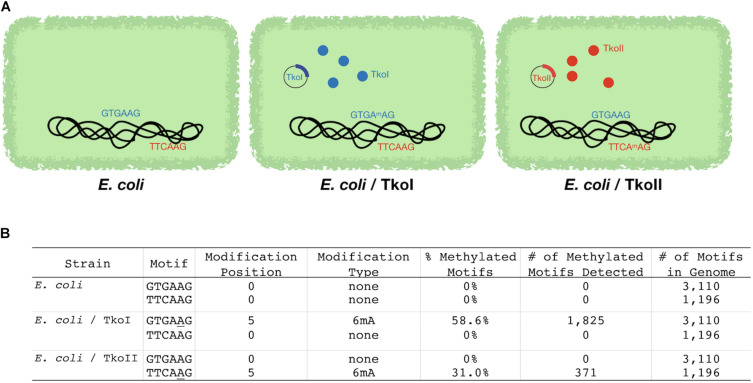
*Escherichia coli* strains expressing TkoI and TkoII demonstrate heterologous MTase activities *in vivo*. *E. coli* was transformed with an empty plasmid or plasmids encoding TkoI (blue) or TkoII (red) R-M systems (schematically illustrated in **A**). **(B)** PacBio SMRT sequencing was used to sequence genomic DNA from each strain. After sequencing, PacBio Modification and Motif software identified sequence motifs that were modified in each strain.

An alternative approach orthogonally confirmed the TkoI and TkoII MTase recognition sites in a heterologous host. Genomic DNAs recovered from *E. coli* transformed with a plasmid expressing either TkoI or TkoII showed evidence of new methylation patterns dependent on the activities of TkoI or TkoII (Figure 3A). The *E. coli* host DNA adenine methylase (dam) methylates the motif GATC throughout the genome and is detected by PacBio Modification and Motif analysis (data not shown). Genomic DNA from *E. coli* strains expressing TkoI were also modified at GTGAAG and strains expressing TkoII were modified at TTCAAG (Figure 3B). Together these data demonstrate that TkoI methylates one strand at GTGAAG sites while TkoII methylates one strand at TTCAAG sites (methylated base is underlined).

### Characterization of TkoI and TkoII *in vitro* Methyltransferase Activity

**FIGURE 4:**
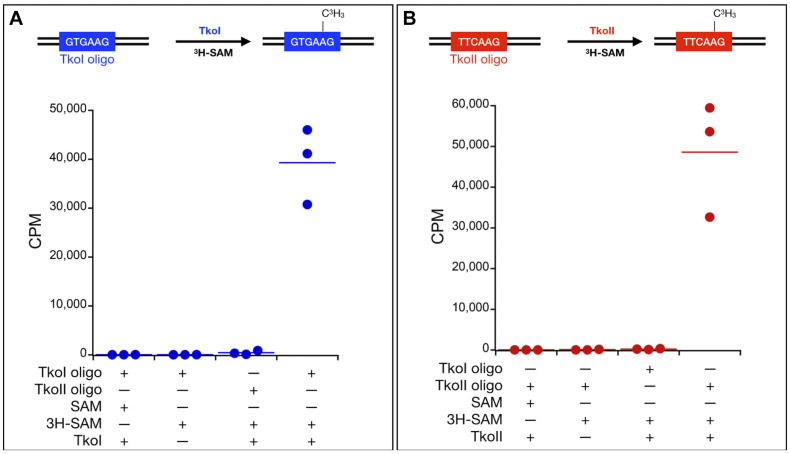
*In vitro* characterization of MTase activities of TkoI and TkoII. Site-specific MTase activities of **(A)** TkoI (blue) and **(B)** TkoII (red) were confirmed by the transfer of ^3^H-methyl groups to DNA substrates (TkoI or TkoII oligo) from ^3^H-methyl SAM (^3^H-SAM). Reactions lacking enzymes or ^3^H-methyl SAM displayed background levels of ^3^H-methyl transfer, as did reactions of TkoI or TkoII with substrate lacking the defined recognition sites. Reactions were completed in triplicate.

Type IIL enzymes typically transfer the methyl group of SAM to DNA substrates containing the recognition sequences of the RM enzyme. The enzymatic transfer of the ^3^H-methyl group from tritiated SAM to a DNA substrate containing the recognition sequence provides an easily monitored and quantified assay of MTase activity *in vitro*. To confirm recognition site-specific MTase activity, TkoI was incubated with a DNA substrate (TkoI oligo, Figure 4A) that contained a single, centrally located GTGAAG recognition site. Transfer of the ^3^H-methyl group to the DNA was robust for TkoI oligo, while only minimal TkoI-dependent transfer of ^3^H-methyl was evident for TkoII oligo, containing a single, centrally located recognition sequence for TkoII (TTCAAG). Substituting TkoII for TkoI yielded efficient ^3^H-methyl transfer to TkoII oligo, while simultaneously reducing methylation of TkoI oligo to near background levels (Figure 4B). No significant transfer of ^3^H-methyl was evident without enzyme addition for either DNA substrate.

### Characterization of TkoI and TkoII *in vitro* Endonuclease Activity

**FIGURE 5:**
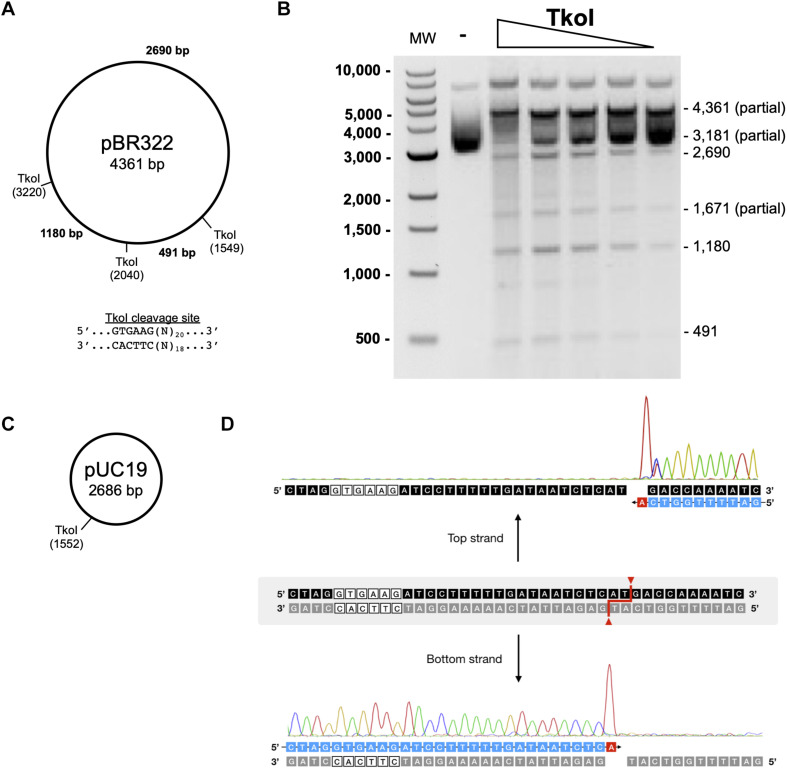
Characterization of TkoI restriction endonuclease activities. **(A)** Predicted TkoI recognition sites in plasmid pBR322 are at positions 1,549, 2,040, and 3,220. **(B)** TkoI was incubated with pBR322 in 1X NEBuffer 3 supplemented with SAM and 90mer trans-DNA for 30 min at 65°C. Reactions were halted with Proteinase K and loading dye and separated on a 1% agarose gel. Lane 1 is a 1 kb DNA ladder. Lane 2 is pBR322 alone and lanes 3–7 are decreasing amounts of TkoI (400–25 nM). **(C)** TkoI cleaves pUC19 at a single site. **(D)** pUC19/TkoI cut fragments were analyzed by run off sequencing to determine TkoI cut sites on the top and bottom strands: GTGAAG(N)_20_/(N)_18_.

**FIGURE 6:**
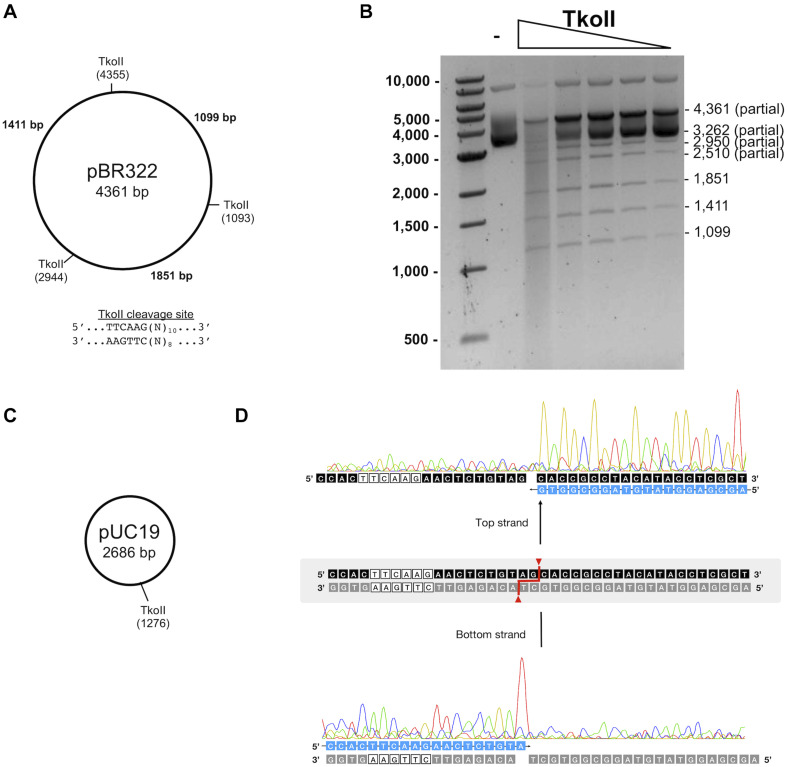
Characterization of TkoII restriction endonuclease activities. **(A)** Predicted TkoII recognition sites in plasmid pBR322 are at positions 1093, 2944 and 4355. **(B)** TkoII was incubated with pBR322 in 1X NEBuffer 1 supplemented with SAM and 90mer trans-DNA for 30 min at 65°C. Reactions were halted with Proteinase K and loading dye and separated on a 1% agarose gel. Lane 1 is a 1 kb DNA ladder. Lane 2 is pBR322 alone and lanes 3–7 are decreasing amounts of TkoII (10–0.62 nM). **(C)** TkoII cleaves pUC19 at a single site. **(D)** pUC19/TkoII cut fragments were analyzed by run off sequencing to determine TkoII cut sites on the top and bottom strands: TTCAAG(N)_10_/(N)_8_.

Standard restriction digestion assays were used to map TkoI and TkoII endonuclease cleavage sites (Figures 5, 6). Both TkoI and TkoII are hyperthermophilic REases that cleave at 65°C and both enzymes would likely function at even higher temperatures. The plasmid substrate pBR322 contains three predicted TkoI sites and three predicted TkoII sites, while the pUC19 plasmid substrate contains only a single predicted TkoI and a single predicted TkoII site (Figures 5, 6). Both TkoI and TkoII cleaved pBR322 and pUC19 with the expected restriction fragments, however, partial digestion also occurred (Figures 5A,B, 6A,B and Supplementary Figure 5). TkoI cleavage activity was similar between 65–85°C and TkoII cleavage activity was similar at 65° and 75°C and decreased at 85°C (Supplementary Figure 6). Both TkoI and TkoII methylate as well as cleave DNA, therefore, partial digestions may fail to go to completion as a result of a proportion of recognition sequences becoming methylated during incubation and thus are resistant to cleavage. TkoI and TkoII cut site specificity was further refined by run off sequencing using the linear pUC19 band (Supplementary Figure 5). As shown by runoff sequencing, the TkoI cleavage site is GTGAAG(N)_20_/(N)_18_ (Figures 5C,D) and the TkoII cleavage site is TTCAAG(N)_10_/(N)_8_ (Figures 6C,D).

### TkoI and TkoII Limit Transformation Efficiencies and Likely Protect Genome Integrity *in vivo*

**FIGURE 7:**
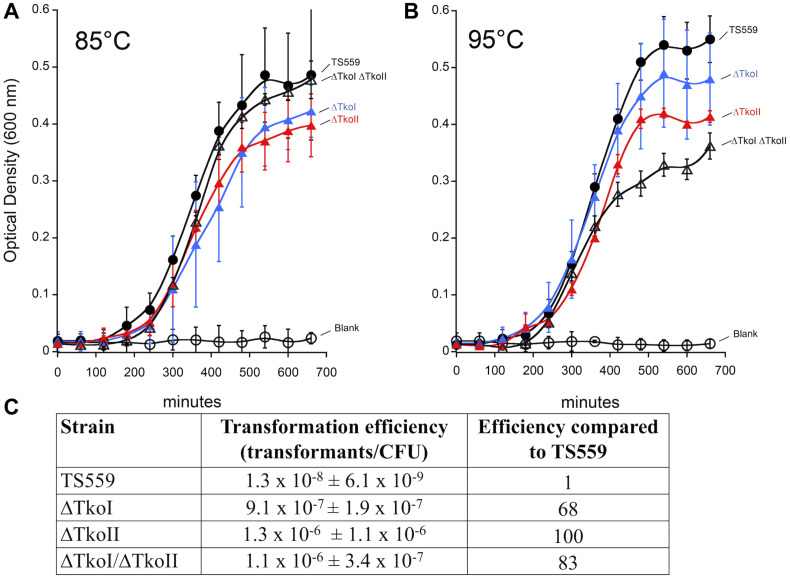
Deletion of TkoI and TkoII impacts transformation efficiencies and growth rates. TS559 (closed circles) and strains ΔTkoI (blue triangles), ΔTkoII (red triangles) and ΔTkoI/ΔTkoII (open triangles) were grown at either **(A)** 85°C optimal growth temperature or **(B)** at 95°C to induce heat stress. Optical density was monitored over time, *n* = ≥9. **(C)** TS559, ΔTkoI, ΔTkoII, and ΔTkoI/ΔTkoII transformation efficiencies (transformants/CFU ± standard deviation) and efficiency compared to TS559 are reported.

To understand the *in vivo* role of TkoI and TkoII we determined if either R-M system was necessary for viability and what impacts might result from deletion of either enzyme. Standard genetic procedures (Gehring et al., 2017) easily permitted deletion of genomic sequences encoding either or both R-M enzymes, suggesting methylation of the genome was not necessary for any cellular processes. Minimal impacts to the growth rate of single (ΔTkoI or ΔTkoII) or double deletion (ΔTkoI/ΔTkoII) strains at optimal growth temperature (85°C) was observed, suggesting that under non-stress conditions, these R-M systems have little impact on cellular fitness (Figure 7); such minimal impacts of R-M systems on phenotypes are common as their role is predicted in preventing exogenous DNA uptake, not general cellular fitness. However, a more pronounced growth defect under heat stress at 95°C suggests that modification of the genome may impact DNA transactions at 95°C as strains lacking one or both R-M systems are less fit than their parental counterparts (Figure 7).

A more pronounced impact of deleting one or both R-M system was predicted on the genetic prowess of *T. kodakarensis*. Previous studies suggested that the transformation efficiency of unmethlyated plasmid was lower compared to modified plasmid DNA perhaps due to restriction by *T. kodakarensis* R-M systems (Fukui et al., 2005). Therefore, if TkoI or TkoII R-M systems restricted foreign DNA, then deleting one or both TkoI and TkoII might permit more efficient transformation of plasmid DNA due to a lack of R-M system mediated cleavage. Transformation of *T. kodakarensis* with the autonomously replicating plasmid pLC71 (Santangelo et al., 2008; Gehring et al., 2017) is easily achieved at low frequencies despite the presence of both active R-M systems and a combined total of nine recognition sequences for TkoI and TkoII; four for TkoI and five for TkoII (Figure 7). Transformation efficiencies increase up to two-orders of magnitude when one or both R-M system is deleted, and perhaps surprisingly, deletion of just a single R-M system was sufficient to boost transformation efficiencies to approximately the same level as deletion of both R-M systems. The increased transformation frequencies of strains deleted for only a single R-M system imply that both systems are imperfect in preventing exogenous DNA uptake and thus helping to explain the retention of so many redundant systems (ABI, BREX, CRISPR-CAS, DISARM, DND, etc.) to protect genomic integrity. Transformation efficiencies of pTP-based replicative vectors (Catchpole et al., 2018), which contain two TkoI sites, and a single TkoII site, into *T. kodakarensis* strains was also improved by deletion of one or both R-M system (data not shown).

## Discussion

The explosion of genome sequencing and the direct identification of modified bases has yielded an incredible number of new sequenced genomes, new predicted pathways and new predicted protein activities. However, biochemical characterization of many proteins has lagged behind bioinformatic predictions and annotations. For examples, even though TK1460 (TkoI) and TK1158 (TkoII) were predicted in the original 2005 *T. kodakarensis* genome sequence to be part of a R-M system, the activities were not characterized until now.

Like other Type IIL restriction-modification systems, both TkoI and TkoII are fused restriction-MTases in a single large polypeptide. *In vivo* genome analysis by SMRT sequencing along with *in vitro* tritiated SAM assays confirmed the MTase activity of these two enzymes is specific for one strand of a recognition site, while *in vitro* REase assays utilizing gel electrophoresis and Sanger run-off sequencing demonstrated the REase activity occurs downstream from the recognition site. Future studies will examine the competition between MTase methylation and REase cleavage on non-methylated DNA by TkoI and TkoII. Further, these enzymes may require other accessory proteins or co-factors to aid in activity that have not yet been identified.

PacBio SMRT sequencing is an important tool that has accelerated identification of new modification MTases by directly detecting modifications during genome sequencing and identifying new motifs (Clark et al., 2012; Murray et al., 2012; Blow et al., 2016). Prolific sequencing of Bacteria and Archaea by PacBio SMRT sequencing has uncovered thousands of putative MTases and restriction-modification systems^1^. Even though PacBio SMRT sequencing enables rapid identification of modification sequence motifs, more biochemical investigation is often needed to assign biochemical function to candidate MTases. In this study, PacBio SMRT sequencing identifies two motifs and two candidate MTases: TkoI and TkoII. Through *T. kodakarensis* strain engineering and over expression of TkoI and TkoII in *E. coli*, we clearly assigned recognition sites for both TkoI (GTGAAG) and TkoII (TTCAAG) by detecting modification motifs with PacBio SMRT sequencing.

Typically, restriction-modification systems are thought to be important as part of defense systems against invading foreign DNA such as mobile elements, plasmids or viruses (Pingoud et al., 2014). Foreign DNA is likely unmodified or modified with a different pattern than the host DNA, thereby allowing cellular REases to cleave and initiate pathways that ultimately destroy invading DNA. In *T. kodakarensis*, methylation of one strand of host genomic DNA by either TkoI or TkoII is sufficient to protect the genomic DNA by blocking endogenous endonuclease activity. TkoI and TkoII cleave unmodified DNA and thus have the potential to cleave invading unmodified foreign DNA leading to a cascade of host enzymes that will degrade the DNA further. Transformation data shows that *T. kodakarensis* strains with intact R-M systems severely restrict transformation with unmodified plasmids DNA (Fukui et al., 2005). In strains lacking TkoI and TkoII, transformation efficiency increases dramatically (∼70–100-fold) suggesting that without TkoI or TkoII foreign DNA is able to survive long enough to efficiently transform more of the population.

Even though this study assigned function to the *T. kodakarensis* restriction-modification systems TkoI and TkoII, future studies are needed to understand the structure and function of these enzymes to define molecular mechanistic details for DNA substrate recognition, methylation and endonuclease cleavage. The noted fitness defects of strains lacking R-M systems suggests that methylation of the *T. kodakarensis* genome may impact DNA transactions at higher temperatures, perhaps by impacting DNA or chromatin structure and thus gene expression, or by influencing DNA replication or repair strategies on a genome-wide or selective portion of the genome. The overrepresentation of recognition sites for TkoI and TkoII within the *T. kodakarensis* genome and the non-uniform distribution of these sites along the *T. kodakarensis* genome suggests the possibility that methylation of the genome by TkoI and TkoII can selectively impact expression of different regulons that support growth at elevated temperatures. The unanticipated differences in growth phenotypes of each strain at normal and elevated temperatures may be explained by the differential impacts of the loss of DNA methylation on the expression of regions of the genome that retain the bulk of recognition sequences for each enzyme. PacBio SMRT sequencing has predicted many more archaeal restriction-systems, therefore, there is a need for further studies of the breadth and diversity of archaeal enzymes. Additional investigations are also needed to explore other archaeal host defenses against foreign DNA. In future studies, it will be important to study other emerging host defense systems (ABI, BREX, CRISPR-CAS, DISARM, DND, GABIJA, HACHIMAN, LAMSSU, SEPTU, Toxin-Antitoxin, WADJET, and ZORYA systems etc.) to understand their relative contribution (Makarova et al., 2013; Zhang et al., 2020). Since hosts are constantly under threat of introduction of foreign DNA, it is likely that many redundant defense systems exist to maintain genome integrity.

## Data Availability Statement

The original contributions presented in the study are included in the article/Supplementary Material, further inquiries can be directed to the corresponding author/s.

## Author Contributions

AFG initiated and conceptualized this study. AFG, RM, and TS guided experimental design. BB generated and phenotyped *T. kodakarensis* strains for this study, and performed transformation efficiency experiments. KZ and AMG performed PacBio library construction, sequencing, and analysis of *T. kodakarensis* strains. KZ expressed and purified RM enzymes. KZ and AFG performed *in vitro* REase experiments. KS performed *in vitro* MTase experiments. KZ, RM, AFG, and TS wrote and edited manuscript. All authors contributed to the article and approved the submitted version.

## Conflict of Interest

The KZ, AMG, RM, and AFG are employed and funded by New England Biolabs, Inc., a manufacturer and vendor of molecular biology reagents, including restriction endonucleases. This affiliation does not affect the authors’ impartiality, objectivity of data generation or its interpretation, adherence to journal standards and policies or availability of data. The remaining authors declare that the research was conducted in the absence of any commercial or financial relationships that could be construed as a potential conflict of interest.

## Footnotes

1 http://rebase.neb.com
