# Supplementary material for: The Hyperthermophilic Restriction-Modification Systems of Thermococcus kodakarensis Protect Genome Integrity
Source: Front Microbiol. 2021 May 20;12:657356. doi: 10.3389/fmicb.2021.657356 (PMC8172983; doi:10.3389/fmicb.2021.657356)
Supplement: Supplementary file 1 [file Data_Sheet_1.PDF]

Supplementary Material

The hyperthermophilic restriction-modification systems of *Thermococcus kodakarensis* protect genome integrity

Kelly M. Zatopek[1], Brett W. Burkhart[2], Richard D. Morgan[1], Alexandra M. Gehring[1], Kristin A. Scott[2], Thomas J. Santangelo[2], Andrew F. Gardner[1]

[1]New England Biolabs, Ipswich, MA, USA

[2]Colorado State University, Department of Biochemistry and Molecular Biology, Fort Collins, CO, USA

1 Supplementary Figures and Tables

1.1 Supplementary Figures

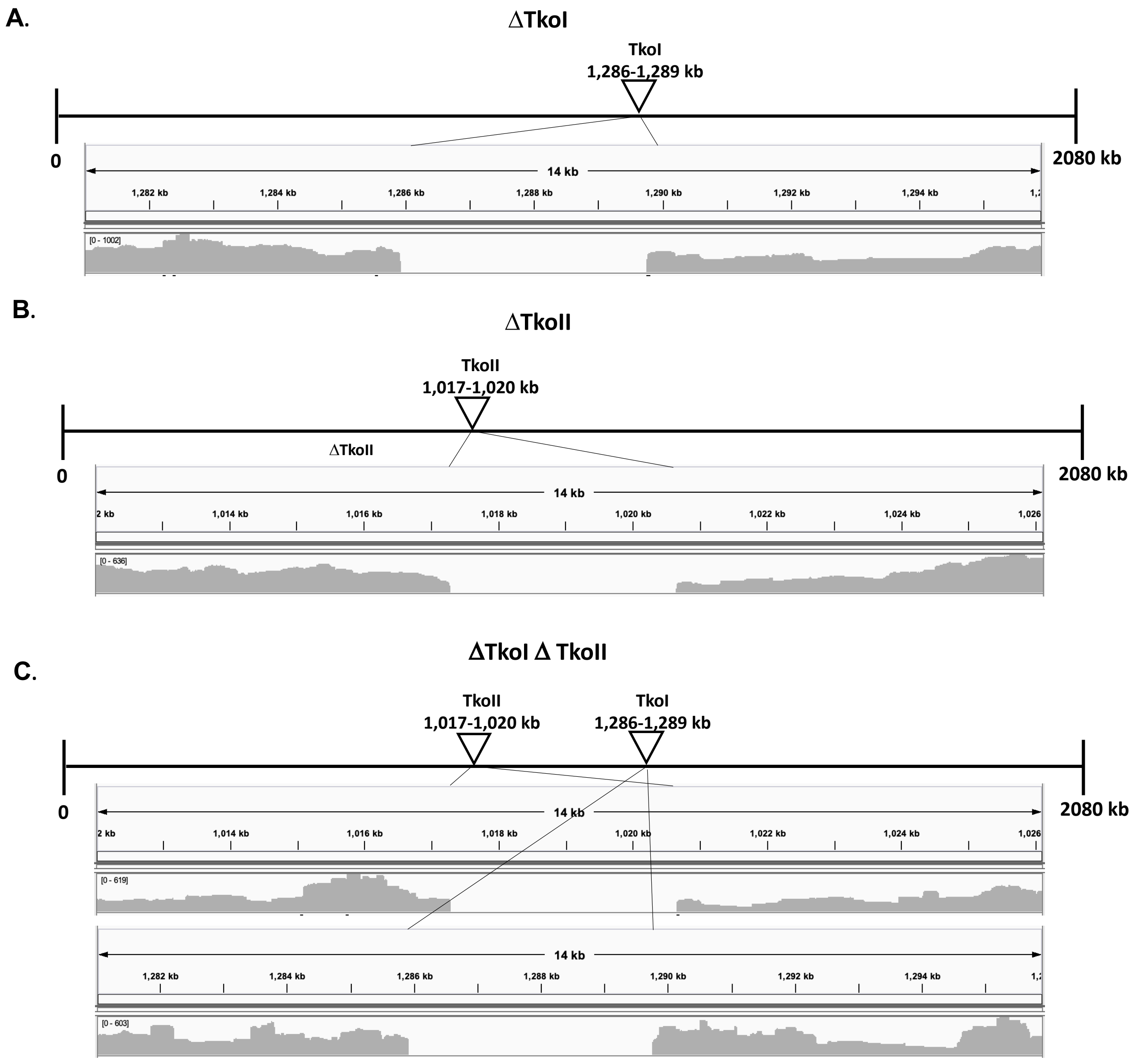

Supplemental Figure 1. PacBio sequencing confirms the genetic deletion of (A) TK1460 (TkoI), (B) TK1158 (TkoII) and (C) TK1460 and TK1158 (TkoI/TkoII). PacBio sequencing reads for libraries generated from *T. kodakarensis* deletions strains were mapped to the reference genome using Integrated Genome Viewer (IGV). The x-axis indicates the *T. kodakarensis* genomic position and the y-axis indicates the fold coverage (~500-fold for each strain) at each position.

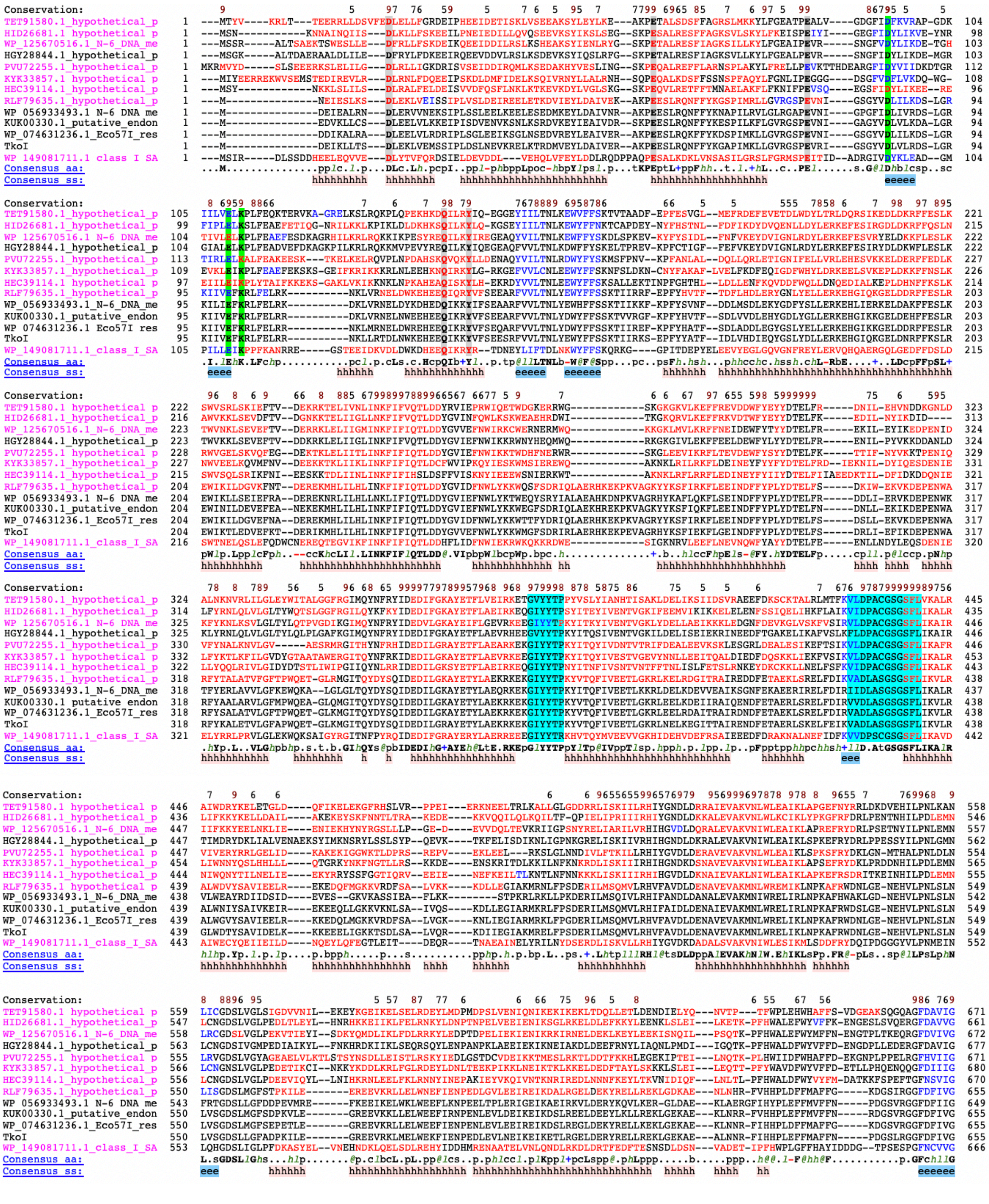

```
Conservation:                       9998        5                     6     9 5 6   6 6  8 5769            7  5                  68  5          5 6
TET91580.1_hypothetical_p    672    NPPYYVEVREH--S------RDFRIFKASPLTG-SYYEAKMDVFYFFVEVGLDLLKIGGNLGFIIMEYWYSRSHAKLLRDRIAHN-SRLYELVHFNEF-KVFEGAK-GQHNDVILLERDEIE   781
HID26681.1_hypothetical_p    662    NPPWISFGLRGTEK------LPQDLQEYLRLTYPNSMEYKGSIYAIFMNLGISKLKNRGIFSFILPDSFLLGRYFSRLRRYILDT-CNIKEISLILRD-FWPDGSV-GRSVIINLQKEN---   771
WP_125670516.1_N-6_DNA_me    673    NPPYIEYRLMV-KK------LGTVYARFLKAIY-KCTQGQFDITIPFTEKSLKLLKKGGILSYIMTNKFMIAEYGYRLRDYLLNS-QVLKEIIDVSNI-KVFKDAA-TYPVIFIVRRVD---   780
HGY28844.1_hypothetical_p    678    NPPYVDSETMT-------RSFP-VLREYCTRSY-ECASGNWDLFCVFVERGIINTKIGGRFGFILPNKLLSADYAEATRGLIKRY--QVEILSDYSRV-KVF-DAA-VYPVVLVLKKNQ-WN   784
PVU72255.1_hypothetical_p    672    NPPYGRIKQLI--KDKT---VKDAFSKYYDSAY-EYQRGNYNYYKLFLERSYKLQRRGGYFSMIFPSSFLGEEDSQPLRKLFFEN-AQVIRILEFPESTEVFGEKA-TQAVTIIVYRKE---   782
KYK33857.1_hypothetical_p    681    NPPYVTLALGK--KQKF---FTDEELKYLREVYPTSSEYKGNTYSLFIDRCTSLCKEKGLLSFIVPNTLLLNTTARRIRKKILDT-FDITLLLNIKT--KVFDSAE-VGGNLVFVFRKG---   790
HEC39114.1_hypothetical_p    671    NPPYFTIRGKG--TGSLVQTYSYSYLQNVPNWK-SHFRSQSDIYYYFIIKSINLLKKGSNFGFIIESYWLENDYADKLKKEIIES-CSLKILINFGRVKKIFEDAD-NDTCILLFEKII-EK   786
RLF79635.1_hypothetical_p    656    NPPYVRIQNLK--K------ENPAYVEFLNRFY-ESSHKNYDLAIPFIERGYSLLRENGELGFIVTKKWMKADYGEKLRGILARE-KAVRLIIDFGDE-QVFKGAT-TYTMILVLRKAK---   762
WP_056933493.1_N-6_DNA_me    650    NPPYVRIQNLK--K------ESKEYVDFLNKFY-ETAHKNYDLAIPFIERGYRLLKDEGELGFIVTKKWMKADYGEKLRELLARE-KAVRLIIDFGDN-QVFKGAT-TYTMILILEKAK---   756
KUK00330.1_putative_endon    656    NPPYVRIQNLK--K------ESPEYVEFLNKFY-ETSYKNYDLAIPFIERGYSLLKENGELGFIVTKKWMKAEYGEKLRKMLAET-KAVRLIIDFGDE-QVFRGAT-TYTMILVLRKAK---   762
WP_074631236.1_Eco57I_res    656    NPPYVRIQNLK--K------ESPEYVEFLNRFY-ESSHKNYDLAIPFIERGYSLLRDNGELGFIVTKKWMKADYGEKMRGILAKE-KAVRLIIDFGDG-QVFKGAT-TYTMILVLRKAK---   762
TkoI                         656    NPPYVRIQNLK--K------ESPEYVEFLNRFY-ESAHKNYDLAIPFIERGYNLLREGGELGFIVTKKWMKIDYGEKLRGLLSRE-RAVRLIIDFGDE-QVFKGAT-TYTMILVLRKER---   762
WP_149081711.1_class_I_SA    667    NPPYVNAINRG--RY-----FSDPEDDFWRWAF-NSADRAFDLYLPFIEQGLNLAVEGGSVSFIVPNKHLAIPYAEAFRDFIVEEDNYLRKITDVSQL-DVFEDPS-VYPVIPHYEKNA---   775
Consensus_aa:                       NPPYhp.......+......bs..h.phh..hb.p..p.p.slhh.Fl-.thpLh+..G.ht@Ih.p.@h..pht..hRcblhcp...lpblhphsp..pVFcsts.s.s.llhl.+.....
Consensus_ss:                                               hhhhhhhhhh               hhhhhhhhhhhhh      eeeeee  hhh     hhhhhhhhhh      eeeeee              eeeeeeeee

Conservation:                                                                                                                  76              5
TET91580.1_hypothetical_p    782    EAEPKEIHVIDV----------LNPS--------LSAAQVHDALLRGEFIEDEIIRRRRILRYDVEQGKFHLRSP-------TVEILIQKIEENQN--FKVEP-ISQG------LVIAIDKV   869
HID26681.1_hypothetical_p    772    KELESRNNKLKAV--------LYEEV--------EDIIEKGYISSYS----YEQDYFENIIH----NRFRLFFEE-------KSKNFVENMEEKGIKLGSRVS-IHTG------VRSKIGQK   855
WP_125670516.1_N-6_DNA_me    781    ENLQYNVRIGSP----------SEKE--------MVEGSPSLTEVPK----SIFTS--ELPL----CILTPYITG-------MHLKLFKKVQSGHAIP--EN-----L------FKCGVART   854
HGY28844.1_hypothetical_p    785    ETDKVDIRIFHE----------------------VPEKAPEITRHTE----LSLNDLYNLPG----NIWSP-AVD-------PALEILNKIRQKSSIM--SEH-YTVL------GGATVSEA   859
PVU72255.1_hypothetical_p    783    KPSPDHAVEIRT----------NIAK--------EELTKLSQ-LYFI----KVKVEDIKRFTGENYRIPMFTNPR-------EELPILEKISKIPPFEGGGGV-PRVGDVYEGELHETQDK-   872
KYK33857.1_hypothetical_p    791    KTKGNKVNLAEV----------KTIQ--------DITSKRFDVKPID----QDEYLQ--------SDDFKFYTEV-------ETLYIVKKMLKNSRKLGEIVD-FYQG------IITGNNKK   868
HEC39114.1_hypothetical_p    787    ENRMKYVYCKKNFSVGTPQLNNRTLI--------AHIIQNISKEAFS----DEYIDIFWVEQNSLKTTKWVLS---------KLTEMITKIEDYKALLGSLCE-IGQG------MVPGRKKE   880
RLF79635.1_hypothetical_p    763    NERLTYAKVEEL----------KENI--------EQLRAVHEPDKWR----EERISVIEVPGDELSEKPWVFLSE-------REREIVKKIYEGSRRLEEVAD-IFVG------VQTSADQV   848
WP_056933493.1_N-6_DNA_me    757    RERFKYAYVKEL----------KESV--------EQLKVVHDAEKAR----RERIQVVEVPMEEISKDVWVILTE-------EEKAIVGKIYKGSKRLEEIAD-IFVG------LQTSADPV   842
KUK00330.1_putative_endon    763    NDKFTYAKVEEL----------KESI--------EQLRAVEESRKWN----EGRLSVLEVPEEELSEKPWVFLTE-------KERKIVEKVYGGSVRLEEVAKHIFVG------VQTSADSV   849
WP_074631236.1_Eco57I_res    763    NERLTYAKVEEL----------KESI--------EQLRAVREPEKWN----EQRLNVIEVPEEELSEKPWVFLTE-------KERKIVKKVHEGSVRLEEVAD-IFVG------VQTSADQV   848
TkoI                         763    NDKLTYAKVEEL----------KETV--------DQLRAVNEPDKWN----SERLSVIKVPTKELSEKPWVFLTE-------EEKKIVEKIYNGSKRLGEVTD-IFVG------LQTSADPV   848
WP_149081711.1_class_I_SA    776    LGS--EATVTLN----------KPPS--------QELLGDEEYNFIH----DHG------TLDKLDENIWSFLLL-------RDTSLFWNIFEGCEQLRSEGT-VQAS------STTSESEE   853
Consensus_aa:                       p.....h.h.p..........p.............h......p.hp..........bpl......p....h..........p...llpKlbp....b...s..h..t......h.s..sp.
Consensus_ss:                           eeeeee                     h         hhhhhhh           h  hhhhh                  hhhhhhhhhhh

Conservation:                                                                                                 5   5
TET91580.1_hypothetical_p    870    TGAILKSLSEKEGDDAIRKYDLREGVFVISPP------ELASMSLNRYEKE-------LIRPYFEAKRVSPYI----INMDNPDSIIYTSKTYIDF-------DDRDVYYEAQKRLKRLDVS   967
HID26681.1_hypothetical_p    856    NI----------------------------------------ISKTKKGTT-------WKRGLISGSEIDRYS----LKYE---------GHFLNI---------------------------   891
WP_125670516.1_N-6_DNA_me    855    -------------------------GFS----------DKIKSSTEAKEGD--------YIRFVQGEDITPF-------YI-----IPPDKYF-----------------------------   892
HGY28844.1_hypothetical_p    860    YE--------------------IGA--------------KLKEIEEATSKE--------FLKFVNTGTIDKYI----SLWG-----FVPTQFI---------------KKS-----------   904
PVU72255.1_hypothetical_p    873    ----------------------------------------AFLSETP-------TGSLVVKGIHLDQYF----VNL------------------------------DPEGPQPRWVR--   908
KYK33857.1_hypothetical_p    869    FL--------------------------------------SHEKIND-------MYKPILRGRDIDRFA----LNFS---------NTYVLF-----------------------------   902
HEC39114.1_hypothetical_p    881    FKISSQ-----------EDIGAGGGYWTSKDDKFFDVVNIKSNTAYRIESL-------FLRPLITNSGIRKFF----IVPS---------KDYLIY------------------TVPFQD--   951
RLF79635.1_hypothetical_p    849    YI--------------------LEL--RGERGNYYLVYSKATNKEYLLEKD-------LLKPLLKNRDTKRWI----ILEY---------KNLLLF-------PYKIVQENGKRKSKIIS--   919
WP_056933493.1_N-6_DNA_me    843    YI--------------------FEL--KEELDQYYRVYSKATNKEYLLEKD-------LLKPLLKGEEIKRWI----VPNY---------KYLLLF-------PYKILKEEGMRKAELLS--   913
KUK00330.1_putative_endon    850    YI--------------------LEY--LGESEGYYIVRSKVTGQEHRLEKA-------LLHPLLKGDEIKRWL----IPEY---------RYLLLF-------PYRITEENGQRRADLIP--   920
WP_074631236.1_Eco57I_res    849    YI--------------------LEL--REERGDYYLVYSKATNREHLLEKN-------LLKPLLKNRDTKRWT----ILEY---------KNLLLF-------PYRIVQENGRRKSKIID--   919
TkoI                         849    YI--------------------LEY--VGESGNYYIVHSKITNQEHRLEKD-------LLHPLLKGEEIRRWM----IPEY---------RYLILF-------PYKVHLVNGERKAELIP--   919
WP_149081711.1_class_I_SA    854    FT------------------------------------IALSEDEPNEN--------KKKFIKTGGVDPFV----STWE-------------------------------------------   884
Consensus_aa:                       h..................................hp.p....pp........hbp.hlps.plc.@h................p.h...........................
Consensus_ss:                                                                       hh

Conservation:                                                                                                                                8
TET91580.1_hypothetical_p    968    EEAKKKTLEVLKKKVRDEILDKVRAKYPTITEHLD--KFRKVITSDR-----------WPYG---------LHRSRDEEIFESS----------------------NKIIGVRKTKF----  1040
HID26681.1_hypothetical_p    892    --------------------------------------------------------EPSIL---------WSGGWDPNVVLN-----------------------NKLLLRQTGDS----   919
WP_125670516.1_N-6_DNA_me    893    --------------------------------------------------------PLS---------LLSEEKITEFKQ-------------------------RKIMIPGIVKD----   918
HGY28844.1_hypothetical_p    905    --------------------------------------YSRPIIST-------------DDLK---------KISEKRLQESRM-------------------------EKIIIAGMCLE----   939
PVU72255.1_hypothetical_p    909    -------------------------------------------------------KKDFL---------KKKPVAEKYVQY-----------------------ERIIGRSTINKELRP   940
KYK33857.1_hypothetical_p    903    --------------------------------------------------------DKEKL---------WSNTDERFFLAD-----------------------EKLINRQTGDA----   930
HEC39114.1_hypothetical_p    952    -------------------GREDINNYPGILAYLK--DNKSELKSRYDYSENPTEQKYPWYG---------YQRIQNIELFETS----------------------KIKILCPYRAEE----  1017
RLF79635.1_hypothetical_p    920    -------------------ENELRTKYPKTWQYLL--ENKELLKNRERGKMKDS---PYWYG---------YIYEKNHDKFEL-----------------------PKAVGKTLANR----   980
WP_056933493.1_N-6_DNA_me    914    -------------------ENELK-KYPKIWEYLN--ENKERLENRERGKLKGS---PRWYG---------YIYEKNHDKFEL-----------------------PKILTGILANK----   973
KUK00330.1_putative_endon    921    -------------------ADELQRDYPRIWEYLNIPEVKKTLEGRDRGAWRGR---ADWYA---------YGRRQNLEVMSV-----------------------PKIVTGVLSDQ----   983
WP_074631236.1_Eco57I_res    920    -------------------ENELRTKYPKTWQYLL--ENRKILENRERGKMRNN---PYWYG---------YIYEKNHDKFEL-----------------------PKAVGKTLANR----   980
TkoI                         920    -------------------IEELASKYPRIWEYLNISEVRKKLEGRERGSLKGS---PKWYS---------YVYEKNHEKFEL-----------------------PKLITGVLSSE----   982
WP_149081711.1_class_I_SA    885    -------------------FEEVRHRGQD--------YEQPVLD-------------TNHE---------IITDLRRDQYNS-----------------------PKLVFAKVSDQ----   928
Consensus_aa:                       ..............................................................h..........h....p..bh.........................Kll....sp.....
Consensus_ss:                                          hhhhhh                                                 hhhh                            eeeee

Conservation:                             6                           65 55799 6   6              6        6   8                        5   5  85          9   5
TET91580.1_hypothetical_p   1041    -PKFVWVPQPCYMAET--------VNYIL--PNKGIDSLYLTTLLNSTLMWFW--FKYGA--EKTHGEQLQIDKEILEMVPLKISDEDV----QMELKDLGNTLGSLCEGENTYLSLWRKWS  1143
HID26681.1_hypothetical_p    920    -LIATLDDTKYYHLNN--------LHSIAP-KNNQYNLKYILALLNSKLLNHY--YHLIS--IELGRAMAQTDIETIEQLPIYPATPDQ----QKPIIELVDKIINLKKSRDQLIAKWNEWS  1023
WP_125670516.1_N-6_DNA_me    919    -IYCGYDTIGMGCGR---------VYYVTE-NESPYPLYYLAALFNSSLFKFIYRVVYGM--SHLSGDYLRINSPYLRIFRVKEIDKEL----ASKFENFVSLLMTLKEAHYKIFKTWREWC  1023
HGY28844.1_hypothetical_p    940    -LECFYDAGEHLAGK---------STVIVL-NGKA-GLKGLVGVLNSQVTSFVLRKLFGS--LALGGGYLRVGPPQVSKLPIPLAFLHSSKE-TKNLENLVDRVEKLKKSRYKFLSFWHEWS  1046
PVU72255.1_hypothetical_p    941    RLKFTILQPGILVTNN--------VKFIVL-TDNKLDKYYVVALLNSSLLNWR--FELFS-------IQNRVSNYDIEELPFYRADPAT----QKAVSDIAKGIIGLKQARYVWFKLWRRAS  1040
KYK33857.1_hypothetical_p    931    -LIAAYDSCKYYTLDS--------THVQVL-KDEGFSLKYILGIFNSKLMDFY--YKTIV--QEEDRPFAQVKTVTLKKIPVKDVPFKQ----QEEIIHLVNKIINLKKLQTTFRDLWYSYS  1034
HEC39114.1_hypothetical_p   1018    -NRFALDNTGYFGTTD--------MYAIVPKSNSKIDLYYLIGILNSKLLTFW--YKEAG---KSKGLILEFFATPLSKMPIVLDGID-----QGTVSKLVLKIVELKKINYRFGQIWFEVS  1120
RLF79635.1_hypothetical_p    981    -STFAIDNSGKFYLT-CGAGGGYGIITKEK-WRGQVSVKFLVASLNSSIVDWR--VKQIA--SEFEGGFYSYDKNAIRNIPIKLPTTDEEKALAKEIEDTVEEIIELLKRHHFVKSLWEEWS  1095
WP_056933493.1_N-6_DNA_me    974    -AKFAPDYEGKFYFVGGGNAGGYGIIPKKE---YDISLTYLTALLNSSLLDWR--LKQLS--TEFEGGFYSYAKRFIEKLPIKLPQTPEEKELAEEIETVTEEIIELLKKHYEIKSLWQKWS  1087
KUK00330.1_putative_endon    984    -ARFSVDLKGDYYFVGGGNAGGYGILIKEE-YKDVVPLKFLVALLNSSLLDWR--LKQLS--TEFEGGFYSYARRFIKDLPIKLPSTDEEKALAEEIEITVDEIIDLLKKHYEVKALWEEWS  1099
WP_074631236.1_Eco57I_res    981    -STFAIDNSGKFYLT-CGAGGGYGIITKEK-WRDQVSVKFLVASLNSSIADWR--VKQIA--SEFEGGFYSYDKNAIRTLPIKLPSTDEEKALVEEIEDTVEEIIDLLKKHHLVRSLWEEWS  1095
TkoI                         983    -PRFALDKNGDYYFVGGGTAGGYGVLIKDD-YKDRISLQFIGALLNSSLLDWR--VKQIG--SEFEGGFYSYGRASIKDLPIKLPSTDEEKTLKEEIEDTVEEIIALLKKHYVVKSLWREWS  1098
WP_149081711.1_class_I_SA    929    -IEAFPDVDGNYASVD--------TNFF---YNSNEEITYYAGLLNSYISNFL--YTGLFGALRMSDGDFQFQAPQLQLIPVPSPETVEDED-IRTVLDIVGRLMDRKHHRERLTTIWTKQA  1035
Consensus_aa:                       ...hhhs....hh...........h.hb....pp.hsbb@lhtlLNSplhp@...h...s...chps.bhph....lp.hPl..s...p.....p.l.phhppl..Lbc.p..h.phW.cht
Consensus_ss:                         eeeee   eee          eeeee           hhhhhhhh  hhhhhh  hhhh           eeeehhhhhh      hhh    hhhhhhhhhhhhhhhhh  hhhhhhhhhhh

Conservation:                          6                       6   6      6                                  7  8          758  9       56  5     55  7 5 7    67 8
TET91580.1_hypothetical_p   1144    STLGNGEISLWDVMAEDQKNLRIGEKG----KTWTKEAS--FFPSNNLGEEQNRLLSE--------EFDNLELS--SSAGHVVIFGA--DNGKT-ELLRIEFENLLMEHIVLSI-HLTLES  1245
HID26681.1_hypothetical_p   1024    TILKN-------------NEYTLHKIL----EEDANHLR--TGTFDKTWTLSATFYPNSENELLNQDFKKFKVIGDNEKSILKIYGLDEQNKEE-LIYEMKFYKREIMQHIYFSI-IALLGS  1124
WP_125670516.1_N-6_DNA_me   1024    TQLKK-------------GEYSLYRIL----SEDARFMQ--MGEFSKAWTSKATFYPTTESKMLNTVFNDFRVVGEVNKHEIKIYGLDENDREK-LVYEMEFSNRELMLHIYCSL-IQALES  1124
HGY28844.1_hypothetical_p   1047    TKLKK-------------SEISFQQIL----TEDMKKFR--EGKFNFVWTSRASFYPNEKNEELKKEYFDFCIVGDVEKNMLRIYGLDENGKGK-LIYEVGFSDRDLMLHVYSSL-IYTLES  1147
PVU72255.1_hypothetical_p   1041    SSLTR-------------DKLSLYEVL----ERDRDAIRTGELDKAWTSYATFYPVNGEQLPEMDRKYWGFMVKGDTRTNTIKIYSL-EKTGFEDPVYELEFRSRELMLHTYSSL-VNTLES  1143
KYK33857.1_hypothetical_p   1035    EKYRT-------------NCKPLKEIL----KEDKRARQ--KGQFDNVWTSDSSVYPDD-SEVLKKEFAEFKVVAEGD-KNLQIYGI-EDSEEV-LVLKVAGRTKEFRDIMYLEL-LALFES  1132
HEC39114.1_hypothetical_p   1121    QNYRN-------------GTITLEKLL----FEDKAKIQ--NDEFEKLWISKINQIPD-----ISKKFKKFKIVIKGK-VGLQIYGI-EGFEEI-RLLDLETTNQEYRDIIYLEI-RQLLQS  1214
RLF79635.1_hypothetical_p   1096    GKLGN-------------KKLTLRKLI----EKWEKGVG--RIPQERLFFTDVKIISD-----EDTEYGGFELE--LKGGSLKLLGR-EGDILT-PVLELE-GKEELLEHIYFSI-LELLES  1187
```

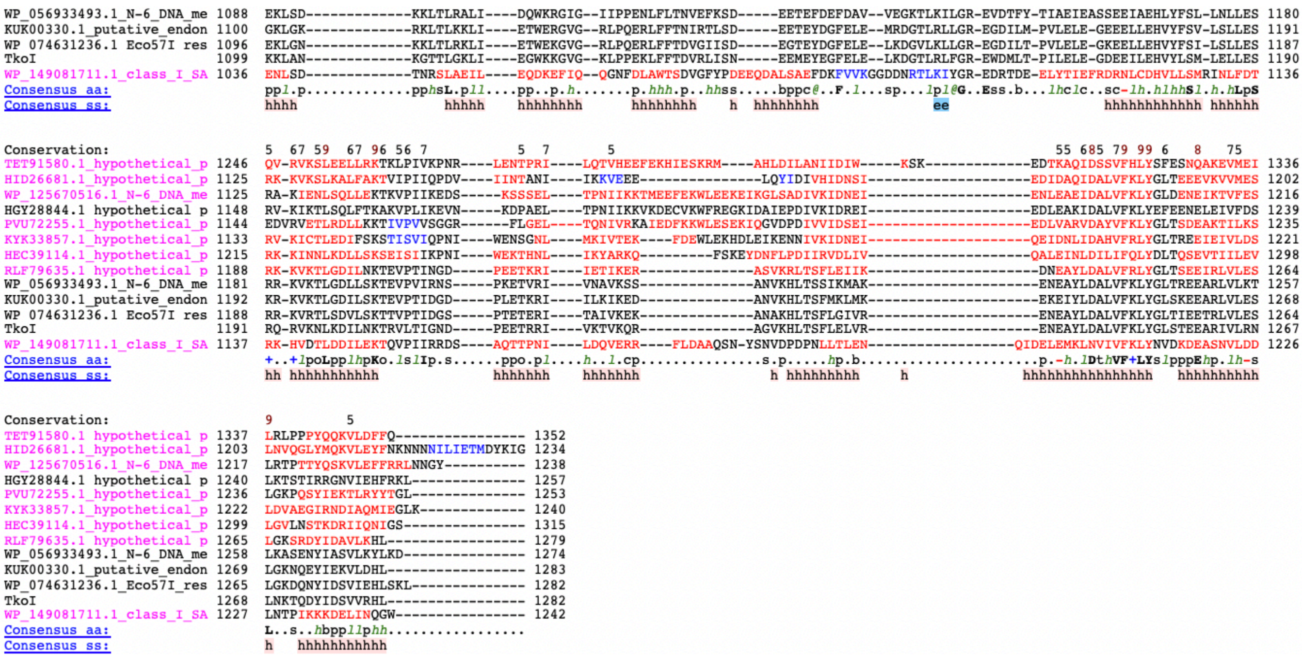

Supplemental Figure 2. PROMALs protein alignment of TkoI and close homologs. The PROMALs multiple sequence alignment server (http://prodata.swmed.edu/promals/promals.php) was used to align TkoI with close homologs and identify the catalytic motifs PD-ExK (green), FxGxG (cyan) and NPPY (yellow). Residues highlighted in red denote predicted alpha helice structure, while residues highlighted in blue denote predicted beta strand structure.

```
Conservation:                                                            6  867         5        5                       9 96566 766977 86 8
NAZ27795.1_class_I_SAM-de    1  ----------------MTAS-----IQLNIDEVLTCIRNAIIQAHTEEDVRVRVSN-CIEERILKPLGITQM-----GKYEYTLI---SGARVDALYRHVVVEYKAPGRLSRDS---DIQ   87
NAZ33036.1_N-6_DNA_methyl    1  ----------------MSSIN----HQIDIDEILTCIRNAIIQAHTEEDVRVRVSN-CIEEKILRPLGITQV-----GKYEYILI---SGARVDALYGHVIIEYKAPGRLSRDS---DIQ   88
AFA38723.1_N-6_DNA_Methyl    1  ----------------MTQVGV---PQIDLDDVLLCIRNSVQKAQSEEDVRVRVSW-CIEEKILKALGLERSA----GKYEYTLV---SGARVDALYGHVIIEYKAPGKLSSAR---DVA   90
WP_011007124.1_class_I_SA    1  ----------------MTQVGI---PQIDLDDVLLCIRRAVQMARSEEDVRVRVSS-CIEEKILKPLGLEHAV----GRYEYTLV---SGARVDALYGHVIIEYKAPGKLSSAS---DVA   90
Nar7I______CTGRAG__1128_a    1  ----------------MPPNSNSL-NQFDIDDILICIRNGILQARTEEDLRIRVSN-CIEEKILKPLNIPFE-----GKYEYTLA---SGKRVDALYGHVIIEYKAPGKLSTDS---DIQ   91
WP_012608870.1_N-6_DNA_me    1  ----------------MSASGTTRVPSLDIDDIVNCFKVHTKKARNEEELRLWVSSYCIEEKILKPLGVLQY-----GKYEYTLV---SGARVDALYGHVIIEYKAPGRLSSEA---DIA   93
WP_012186110.1_N-6_DNA_me    1  ---------------MAAQ------PQISIDDVLNCFLQGIREANNEEELRIRISR-CIEDMILEPLGITQYG----GRYEYTLV---SGARVDALYGHVIIEYKAPGRLSTNT---DIT   88
HGN90406.1_class_I_SAM-de    1  -----------------MS------TGLDLDDVVTSIRDAVSRAQSEEDVRVRVSG-VIEQKILQPLGINSV-----GRYEYTLV---SGVRIDALYGHVLIEYKAPGKLSNKS---DIA   85
WP_125672489.1_N-6_DNA_me    1  -----------------MD------MQVDVDKVLAELKAIAAEAKNEEEFKINAER-VLYNEVISKLGLQP------GRFEYTFV---SGGRADALYGHLIIEYKAPGKLSRPQ---DIA   84
RLG60388.1_class_I_SAM-de    1  -----------------MV------EQVDIERVLTGLNNAARRASTEEEFKIHAER-ILYEEVISKLGLQP------GKHEYTLI---SGVRPDTLYGHLIIEYKAPGKLSKAS---DVA   84
NQE06103.1_hypothetical_p    1  ----------------------------------------MKKASNEEDLKIKVES-WIQ-EVISKFFEQGD-EPEVAYEHRTKI---SGKREDALYGTVIIEYKAPKKLDTRS---EFV   71
RZN40535.1_class_I_SAM-de    1  ----------------MEKPDKGLDLGLEAKRIKGKIKEYARKAGNEEELKIKVEG-LIQ-EIIAKFFPEGE-EPEVAYEHRTTI---SGKRKDALYGTVIIEYKAPRRLDTGN---EFV   95
PXF53089.1_SAM-dependent     1  ----------------MNNSPEL--EKINAQELAEHIKERAQEAGNEEELKIGFAV-LLD-PFLRAWNIKPAYERHAG-GVRCVV---SGVRKDALYGTVIVEFKAPGKLKSEK---EFS   93
TkoII                        1  -----------------ML-------LENLEQVYERIKLAAKQASNEEELRHNVSK-VLD-EIAREFGVSGRLEKSTQLLESKKV---FRGRIDALYGGVIIEYKSPGKLKEKQ---AFN   88
HDJ25919.1_class_I_SAM-de    1  ----------------MAKREGL--EGVDAEELAVQIRQAAEESATEEDLRVRVEH-ILRLKALDKLDVAFG------KYERATVVCGARRRPDALYGYLVIEYKAPGVLSTKQ---GRE   92
TsoI______TARCCA__1116_aa    1  ----------------MSPSREEV-VAHYADRLHQVLQKTIAQNPNEAEFRRAVEP-LLE-EFLREMGLEPL------ARAEYTL---AQGRADAIFNRLVIEYERPGVLKPKPD-AATR   91
TspDTI______ATGAA__1112_aa   1  ----------------MSPSREEV-VAHYADRLHQVLQKTIAQNPNEAEFRRAVEP-LLE-EFLREMGLEPL------ARAEYTL---AQGRADAIFNRLVIEYERPGVLKPRPD-AATR   91
TthHB27I__CAARCA___AAS822    1  MLSLLTGGVFRRVKLMNWIDLYTH-LKQEVPWFFNSVRLAASQAHNEAEFESRINN-AIE-RLAQKLGVQLL------FREQYTL---ATGRADAVYNRLVIEYEPPGSLRPNLKHSHTQ  108
Tth111II__CAARCA__AAU2150    1  ---------------MNWIDLYTH-LKQEVPWFFNSVRLAASQAHNEAEFESRINN-AIE-RLAQKLGVQLL------FREQYTL---ATGRADAVYNRLVIEYEPPGSLRPNLKHSHTQ   93
HauII_____TGGCCA__1093_aa    1  -------------------------------------MYAQRVANFVQEYQRYINN-SAN-EEELRSGFYLAATNALG-IPNFTL---ERGRQDIRRNRVILEFKNKGLFRGQSTSLKFK   77
Consensus aa:                   ............................hphcclh..lp.hh.pA.sEp-hc..lp..hlp.chhp.hsh............hhl...t..R.DAl@spllEYKsPG.Lp.......h.
Consensus ss:                                                hhhhhhhhhhhhhh   hhhhhhhhhh hhh hhhhhh                        ee    eeeeee           hhh

Conservation:                   8   8    97   7         5 97  57 6 966          6     5          55 66 68  65 7    6  887  6  5      66  8         55  9
NAZ27795.1_class_I_SAM-de   88  RAKEQVI-RYIMSEAGGKE-SWDRYLGIIISD-RIAFVRYDKG--SDAWVLR-GPYEIRREVIIKLIEALRGL-RRKSLSVSNIVTDFGPSSPIAKRTVKLLYTKLLSS---GSGRTRLL  197
NAZ33036.1_N-6_DNA_methyl   89  KAKEQVI-RYIMSEAGSKE-LWDRYLGVIISD-RIAFVRYDRS--GGAWILR-GPYEVRREVIIKLIEALRGL-KRKSLSVNNIVADFGPDSPIAKKTIKLMYTKLVNT---KSMRTLIL  198
AFA38723.1_N-6_DNA_Methyl   91  KAKEQVV-RYITEEAGGKS-EWPRYLGVIISD-RIAFVKYDPR--GGDWILR-GPYDIRREVVIKLIEALRGL-RRKNLDVNNLVKDFGPNSPIAMKTIKLLYNKLRSS---NTPRVKVL  200
WP_011007124.1_class_I_SA   91  KAKEQVV-RYIMGEAGSKS-EWPRYLGVIISD-RIAFVKYDPQ--SGTWILR-GPYDIRREVVIKLIEALRGL-RRKNLDVNNLVKDFGPNSPIAMKTIKLLYRKLTSS---NNPRVKVL  200
Nar7I______CTGRAG__1128_a   92  KVKEQAI-KYIITEAGDKS-NYDKFLGIIISD-KIAFVRYDKR--NNIWILR-GPYEIRRESIIKLIEALRGL-RRKILSAEEIAKDFGMNSQISKRSIKLLYTKLLNT---KNEKTKIL  201
WP_012608870.1_N-6_DNA_me   94  RAKEQVI-KYIQQEASSEA-EWDRYLGVIISD-KIAFVRYDKA--GRVWILR-GSYEITRESVIKLIEAIRGL-RRKALRVDLLLDDFGLKSNLTARSVRVLYEKLLKP---KSEKTKVL  203
WP_012186110.1_N-6_DNA_me   89  KAKDQVI-NYIKTEAASKA-EWDRYLGIIISD-RIAFVRYYKP--QDTWILR-GPYNITRESIIKIIEALRGL-RRKALSVDNLLRDFGSQSELAGRVIRALYRRLIET---GNPRTRVL  198
HGN90406.1_class_I_SAM-de   86  NAKAQLV-RYIEKEAEVEN-RYGNFLGVIISD-RIGFLRYNPRASGDKWVLR-GPYDINRETVVKLVEALRGL-QRKSLNVESLVRDFGPQSDVAKKMVRLLYERLMGS---KSARVRVL  197
WP_125672489.1_N-6_DNA_me   85  SAKEQLI-GYIKKEAEVEG-RYKLFLGVILCD-RIAFVRYDPK--TKDWLLR-GPYDLNRESILRLIEAIRGL-RRKKLRTEELLEDFGPKSATTRKAVKVLYNKLLTS---KNPKVEAL  194
RLG60388.1_class_I_SAM-de   85  KAKEQLV-GYLKEEAEVEQ-RYKLFLGVILSD-KIAFVRYDAK--TKDWVLR-GPYDLNRETVLRLVEAIRGL-RKKKLAVKELLDDFGPKSDIAKKAVRVFYKKVTKS---KSVKVEAL  194
NQE06103.1_hypothetical_p   72  KAKKQII-EYINEEAGGKPENFGKFFGVILDGYKISFVRFRR----NQWMAN-EPTELSEESVYRLLEAIISL-RRKAIDADFLLTDFGPESETSEKVISVLYEALEKS---KSSRTEML  181
RZN40535.1_class_I_SAM-de   96  KAKEQVV-EYIKEEAEGAAENFGKFFGVILDGYKISFVKFRR----NEWVAN-EPTEVSEESVYRLLEAIISL-RRKAIDADFLLADFGPESETSEKVISILCAALEKS---TSSRTSML  205
PXF53089.1_SAM-dependent_   94  KAKEQVK-RYIETEAVDPR-YYGRYFGVVLDGYQIAFVRFRK----GDWEEQEKPLEINAYAILRFLEAIRGL-KRKPIDAQLLLEDFGPKSAISKKIILSLYKALAKP---QSSRTEML  203
TkoII                       89  SALDQVI-NYIKSEAKSEE-FYPYYLGVLFDGSTIAFVRYWG----GSFKVN-GPLPFTFEAFEHLVDALRGL-TRKPLDAEMLLRDFGPKSRITEEVLRAFYSALESP---KSDRTEML  197
HDJ25919.1_class_I_SAM-de   93  DAINKLR-IYIEALSRERREDKRRFLGVAVDGHTIIFVRFRA----GRWEER--ALPVNSASVAMWLQAFTGL-SRRALSPDMIVEGLGPKSPVARRLIEVLYRKLVDHGR-TDPRVRAL  203
TsoI______TARCCA__1116_aa   92  HAVQQVK-DYLSGIAQRERHAKERLAGVAFDGRYLIFVRHMG----ERWVEE-PPVEANPHSLKRFLTWLAGLASGIALTSENLNRDFSIEQLRTQTILRGLYQALEKALA-EEGLVRQL  204
TspDTI_____ATGAA__1112_aa   92  HAVQQVK-DYLSGIAQRERHAKERLAGVAFDGRYLIFVRHMG----ERWVEE-PPVEANPHSLKRFLTWLAGLASGIALTSENLNRDFSIEQLRTQTILRGLYQALEKALA-RKGLVRQL  204
TthHB27I__CAARCA___AAS822  109  HAVRQVM-NYIEELSRAERHDRDRLLGVVFDGHYFIFVRYHE----GHWIVE-EPLEVNPASCERFLRSLFSLSSGRALIPENLVEDFGSQNDLSRQATRALYHALQGH---TSDLTARL  219
Tth111II__CAARCA__AAU2150   94  HAVRQVM-NYIEELSRAERHDRDRLLGVVFDGHYFIFVRYHE----GHWIVE-EPLEVNPASCERFLRSLFSLSSGRALIPENLVEDFGSQNDLSRQATRALYHALQGH---TSDLTARL  204
HauII_____TGGCCA__1093_aa   78  EAQDQLINRYIPQQSLRDGRRPSDYIGVCFDGEHLAFTFVEP---DQTVRVT-KLLPFDEHSAGALVMALDVD-DRRELTPQNVIDDFGPSSFIARQTLQALWHHLNVSLDMGVKRIHML  192
Consensus aa:                   cAbpQl..pYI..bt..c.....c@hGVhhss.plhFV+@.......pW..p..sh.hs..sh.+hlpAl.tL.p++.Lssp.llcDFGspS.hspphlphLYp.L..s...ps.+hchL
Consensus ss:                   hhhhhhh hhhhhh          eeeeee    eeeeee        eee        hhhhhhhhhhhh         hhhhhhhh    hhhhhhhhhhhhhhh      hhhhhh

Conservation:                   9 69 6 9 7   6           58     5   57        5   6 855799667  9875 75             6    5  5               5  69586 75
NAZ27795.1_class_I_SAM-de  198  FEDWMRLFKQATGYDP--------GKLKELKELVEEYGLS--ESNVNYDALIFSIHTYYALIMKLIAAEVAYLY--GSGRFYRSYIAELDDAYTK---NSVEGLVAVLRELEDGGVFRRL  302
NAZ33036.1_N-6_DNA_methyl  199  FEDWMRLFKQATGYDP--------NKLKELKKLVEEYGLS--ESNVNYDALIFSVHTYYALIMKLLAAEVAYLY--GSGKFYRSYVAELDDAHAK---RGVEGVAAVLKELEDGGIFKKL  303
AFA38723.1_N-6_DNA_Methyl  201  FDDWMRLFKQATGYDP--------GKLKELPRLAQEYGLT---GEVDYDALLFAIHTYYALIMKLLAAEIAYLY--GGGKFYSSYVAKLDDAYSR---EGVDGIMQILRDLESGGVFRNL  304
WP_011007124.1_class_I_SA  201  FDDWMRLFKQATGYNP--------EELEELPKLAEEYGLT---GGVNYDALLFAIHTYYALIMKLIAAEIAYLY--GGGKFYRSYVAELDDAYSR---EGVEGIRQVLRDLESGGVFRNL  304
Nar7I______CTGRAG__1128_a  202  FEDWTRLFKQATGYDP--------NKFKELKELAEDYGFE--KSNINYDILIFAIHTYYALIMKLIAAEIAYLY--GSGRFYKSYIVELDNAYTS---KGIEGLKNILKELESGGIFKKL  306
WP_012608870.1_N-6_DNA_me  204  FEDWLRLFKQATGYSA--------ERLEELPRLAREYGLT---GEVDYDKLLFAVQTYYALILKLLAAEVVYLY--GGGRFYKSYIAELDDAYTG---GGVQALRKALAKLESGGIFKQ-  306
WP_012186110.1_N-6_DNA_me  199  FEDWMRLFRQATGYRP--------EELEELPTLAQEYGLA---DNVNFDALIFAIQTYYAFILKLLAAEVVYLY--GGGRFYISYIADLDDAYTR---GSVNALRDELRELESGGIFRH-  301
HGN90406.1_class_I_SAM-de  198  FEDWFRLFSQTTGYSP--------DKLRELKRLVEEYGFR---GKVDYNFVIFSLHTYYALVMKLLAAEIAYLY--GAGRWLKSYVGELENAYLR---GGVDSLKTVLRELEEGGVFANL  301
WP_125672489.1_N-6_DNA_me  195  FNDWKRVFSQVCAYSP--------DKLKG---LEAEYEVSN---PVNYDALLFSIHTYYAFFMKLLAAEIAYLY--GAGRFLKSYVAELEDAYI----KGLDFLKSSLTELESGGIFRNL  294
RLG60388.1_class_I_SAM-de  195  FNDWKRLFSQVCAYSP--------EKLEG---LEAQYEMSK---PVDYNALLFSIHTYYAFLMKLLGAEVAYLY--GAGRWLKSYVSELEDAHM----RGLEALKRALEELESGGIFRSL  294
NQE06103.1_hypothetical_p  182  FRDWKRVFSQVCAYSP--------SKLEG---LIEHYGVAKGKKNVDVEKLMFAVHTYYTLVMKLLTSEVISFF----NPVFGSPLQRIESAYY----RSREDLKEELRDLEEGGIIAKI  282
RZN40535.1_class_I_SAM-de  206  FKDWRRVFSQVCAYSP--------SKLEG---LVEHYGV-AKGKSVDVEMLMYAVHTYYTLVMKLLTSEVISYF----NPVFGSPLQRIENAYY----RSREELKDELLDLEEGGIIAKI  305
PXF53089.1_SAM-dependent_  204  FEDWKRVFSQVCAYSK--------DKLKG---LIGYYGLSG---DIDVEKLMFAIHTYYTLLMKLLTSEIVTLF--ADS-LIGSYLKRIEDARM----RGGDEMLLEMEELEEGGIFKEL  302
TkoII                      198  FEDWRRIFSQVCSYTP--------EKLKK---LAEFYGFK----EADPERLTFALHTYYTLLMKLIVSEIVSVL--SEG-LTGSVLARLNESYL----SDVESFRFVLTELEDGGLFRQL  295
HDJ25919.1_class_I_SAM-de  204  FEDWKRVFSQVCAYKP--------EKLRG---LEKEYGIKG---KVNCEALLFAIHTYYALVMKLIAAEVAVLC--TAP-ILRSFFGRLEEAYVAEGDEEKLAFREEFKRLEDGERFRQV  306
TsoI______TARCCA__1116_aa  205  FEQWRIFFSEAIDYSETFG-GRKLEPLKK---WVRKAGL-HIQTPEEAERFFFVLHTYFALLAKLLAWLALSRH--MGV-RLGAP--VFSALAA----ADGETLQKRLGEMESGGIFRQY  310
TspDTI_____ATGAA__1112_aa  205  FEQWRIFFSEAIDYSETFG-GRKLEPLKK---WVRKAGL-DIQTPEEAERFFFVLHTYFALLAKLLAWLALSRH--MGV-RLGAP--VFSALAA----ADGETLQKRLGEMESGGIFRQY  310
TthHB27I__CAARCA___AAS822  220  FVQWQIFFGETAGADAAGGELKHKSELLA---FARGMGLR--GSRIDMPRFLFALHTYFSFLVKNIARLVLQAY--AGG-GLGTT--PLTTIAN----LEGEALRRELQNLESGGLFRTL  325
Tth111II__CAARCA__AAU2150  205  FVQWQIFFGETAGADAAGGELKHKSELLA---FARGMGLR--GSRIDMPRFLFALHTYFSFLVKNIARLVLQAY--AGG-GLGTT--PLTTIAN----LEGEALRRELQNLESGGLFRTL  310
HauII_____TGGCCA__1093_aa  193  FTEWKSLFQQGTSIGTR-----GRQKLQA---YLQSVGLPE---SADITRLLFVLHTYHALFFKLLAAEVVLTNAIIPG-MTQTDF-CFSTAGL----PDRSLIKLLVDDIEESKVFRRV  295
Consensus aa:                   Fp-W.plFpQhhthss........pcLp....hhpphGl......hshp.lhFslHTY@hhlhKLlhtElh.hh...ss.hh.o.h..hpsthh.....s.p.lp..l.-lEpGGlF+pl
Consensus ss:                   hhhhhhhhhhh    h        hhhhh   hhhhh              hhhhhhhhhhhhhhhhhhhhhhhhhhh                hhhhhhhhhhhhhh       hhhhhhhhhhhh    hhhh

Conservation:                    75 97597879599555    5   5      8  9  565 6 85 569659 895 8 9  599 9997799899865 8 556                   5   7 99989999
NAZ27795.1_class_I_SAM-de  303  LNIENFLEGDYFSWYLEEVDRDLADAIAEIARVLSDYEIATPQLEPEFARDLLKRLYQNLVPRDIRHSLGEYYTPDWLAELMLNEVGLSLENLMKMG--EEDSLKPL-RIKVLDPACGSG  419
NAZ33036.1_N-6_DNA_methyl  304  LNIENFLEGDYFSWYLEELDRDLADAIAEIARRLSDYEIATPQLEPEFARDLLKRLYQNLMPRDIRHSLGEYYTPDWLAELMLDEVGLSLEILNRMG--EGDPLKPL-KLKVLDPACGSG  420
AFA38723.1_N-6_DNA_Methyl  305  LNIENFLEGDYFSWYLEVLDKDLADAIAEIARRLSDYEVAAPQLEPEFARDLLKRLYQNLVPRDIRHNLGEYYTPDWLAELVLDEVGLSLDNLLKMG--EEDPLKPL-RIRVLDPACGSG  421
WP_011007124.1_class_I_SA  305  LNIENFLEGDYFSWYLDELDKELADAVAEIARRLSDYEVAAPQLEPEFARDLLKRLYQNLVPGEIRHKLGEYYTPDWLAELVLDEVGLSLKNLLKMG--EEDPLKPL-KIRVLDPACGSG  421
Nar7I______CTGRAG__1128_a  307  LGIENFLEGDYFSWYVEEIDQELGDVIAEIARRLSDYEIATPQLEPEFARDLLKKLYQNLVPRDIRHNLGEYYTPDWLAELIINEVGLSYENLENMG--KDDPLKPL-EIRVLDPACGSG  423
WP_012608870.1_N-6_DNA_me  307  FGIENFLEGDYFSWYLEELDQILADVLAEIARKLANYEPATPQLEPESARDLLKRLYQHLVPEDVRHNLGEYYTPDWLADFLLDRVGLSRDRLNELG--SEYSLRPL-EIRVLDPACGSG  423
WP_012186110.1_N-6_DNA_me  302  FGYENFLEGDYFSWYLEELDGELAGALAEVIRRLSDYEPATPQLEPEYARDLLKRLYQELMPRDIRHNLGEYYTPDWLADFLLDEVGLSLGNLMEMG--KEDSLKPLQQLRVLDPACGSG  419
HGN90406.1_class_I_SAM-de  302  LNIVNFIEGDYFSWYLDVLDKDLGDAVAEVARRLSDYEPATPQLEPELARDLLKRLYESLIPRDVRHKLGEFYTPDWLAELVLDEVGLTAERFEELG--AENSLRPL-ELRVLDPACGSG  418
WP_125672489.1_N-6_DNA_me  295  LNITNFIEGDYFSWYLEELDKELADALCDVAKKLSDYEPATPVLEPELTRDMLKRLYQNLVPRKIRHDLGEYYTPDWLAELVLDEVELTLENFEKLAKERGDNLAPL-SLRILDPACGSG  413
RLG60388.1_class_I_SAM-de  295  LNITNFIEGDYFSWYLEELDEELADVIAEIAKRFADYEPATPVLEPEYTRDLLKRLYQHLVPKKIRHDLGEYYTPDWLADLVLNEVGFTVENFEKLAKERDDALAPL-TLRVLDPACGSG  413
NQE06103.1_hypothetical_p  283  -GIRNFLEADYFAWYLDEWNEDVVKGVIEIVRTLGDYDPATVELDPDRVKDLFKQLYQNLVPKRVRHDLGEYFTPDWLAELVLKEAEY---------------DGNL-DKRVLDPACGSG  385
RZN40535.1_class_I_SAM-de  306  -GIRNFLEADYFAWYLDEWSEDVVAGVMDIVKKLSEYDPATVELDPDRVKDLFKRLYQNLVPKQVRHDLGEYFTPDWLAELVLKEVEY---------------DGNV-EKRVLDPACGSG  408
PXF53089.1_SAM-dependent   303  -GIINFLEADYFAWYIDEWNEEIASLIYEMAKELLDYEPATVELTPERVKDLFKRLYQNLVPREIRHKLGEYFTPDWLAELLLDEVGY---------------DGNP-ENRVLDPACGSG  405
TkoII                      296  -GIRNFLEADYFAWYLEEWSGEVARAVYEITKALMDYEPATVEQTPERVEDLFKRLYQNLVPREIRHSLGEYFTPDWLAELTLDEAGY---------------DGNP-EKRVLDPACGSG  398
HDJ25919.1_class_I_SAM-de  307  -GVVNFTEADYFAWYLHVWDRELAEAIWHLVRVLSDYEIGTVELEPGYVRDLFKRLYQRLVPKKVRHDLGEYYTPDWLAELVLDEVGFTVERLSELAR-EKGPETPF-SLRLLDPACGSG  423
TsoI______TARCCA__1116_aa  311  -GILNLLEGDFFAWYLHAWSSEVERALRALIERLDEYDPTTLSLFPEETRDLFKKLYHYLLPREIRHNLGEYYTPDWLAWRLLVQLDNTFFAGTPSPNDEKLRQKLL-STRFLDPACGSG  428
TspDTI_____ATGAA__1112_aa  311  -GILNLLEGDFFAWYLHAWSSEVERALRALIERLDEYDPTTLSLFPEETRDLFKKLYHYLLPREIRHNLGEYYTPDWLAWRLLVQLDNTFFAGTPSPNDEKLRQKLL-STRFLDPACGSG  428
TthHB27I__CAARCA___AAS822  326  -GLKNLLEGDFFAWYLDAWNPEVEEALRQVLARLAEYNPATVQDDPHSARDLLKKLYHYLLPRDIRHDLGEFYTPDWLAERLLNQLGEPWFIMPPG---NHPPRGLP-DKRLLDPACGSG  440
Tth111II__CAARCA__AAU2150  311  -GLKNLLEGDFFAWYLDAWNPEVEEALRQVLARLAEYNPATVQDDPHSARDLLKKLYHYLLPRDIRHDLGEFYTPDWLAERLLNQLGEPWFIMPPG---NHPPRGLP-DKRLLDPACGSG  425
HauII_____TGGCCA__1093_aa  296  -NILNFVEGTFFTWYTHEAPAGLIEAIRQIMQRLNLYRLSD--LQLERTRDIVKYVYEQIVPEPLRHSLGEYFTPEWLVEFTLDRVGYQ-------------GSQIL-DQKILDPCCGSG  398
Consensus aa:                   .sI.NFlEtD@FtWYlc.hs.-l.ctl.plhppLs-Yc.tTspLpP-.h+DLhK+LYppLlP+clRHsLGEY@TPDWLA-hhLsclshs.............s...h.pbRlLDPACGSG
Consensus ss:                       hhhhhhhhhhhhhhhhhhhhhhhhhhhhhhhhhhh          hhhhhhhhhhhh hhh          hhhhhhhhhhh hhhhhh                      eeee
```

```
Conservation:                    8966 56 6     5                       5769599999986778 9977  5556       77997969985 55                              5  6 7 58
NAZ27795.1_class_I_SAM-de   420  TFIVLYISRLRRYAEEHF-----LVDALPNYVLENVVGYDLNPLAVLTARTNYLLAIADLLPNVR-GSVEIPIYLADSIMIEERY-ELKG-----GTDVYMLRTVAGEFKIPKDVAE-KP  526
NAZ33036.1_N-6_DNA_methyl   421  TFLVLYISRLRRYAEEHF-----LVDALPNYVLENVVGYDLNPLAVLTARTNYLLAIADLLAYVR-GTVEIPIYLADSIMIEERY-ELKG-----GKDVYVLRTVAGEFRIPKDIAR-NP  527
AFA38723.1_N-6_DNA_Methyl   422  TFLILYLSRLRRYAEEHY-----LTDQLLSYVLDNVVGYDLNPLAVLAARTNYLLAVADLLSYAK-GRVEIPIYLADSIMIERNS-KLTG-------EVYVLRSVVGEFQIPRGVVD--K  525
WP_011007124.1_class_I_SA   422  TFLMLYISRLRRYAEEHY-----MTDQLLSYVLENVVGYDLNPLAVLTARTNYLLAVADLLSYAK-GRVEIPIYLADSIMIERNS-HLAG-------DVYLLRSVVGEFQIPRSVVE--K  525
Nar7I______CTGRAG__1128_a   424  TFLILYISRLIRYANEHY-----LTDTLAKYILENVVGYDLNPLAVLTARTNYLLTIADLLQQSGLGSIEIPIYLADSIMIETKA-EITE-------KYYILRTAVGEFKIPVNTAQ-NT  529
WP_012608870.1_N-6_DNA_me   424  TFLVRYIARLRDYAREHF-----LEDVMLDYLLENIVGYDLNPLAVLAARTNYLLMIADLPRR---GSIEIPVYLADSIMVEKRA-TLTA-------NVYVLKTVAGEFQLPVSVVE--K  525
WP_012186110.1_N-6_DNA_me   420  TFLVRYIARLRAYAREYF-----LEDVLVDYVLQNVVGYDLNPLAVLTARTNYLLMIADLPKK---GTIEIPIYMADSLMVERRS-TLMG-------DVYALRTTAGEFRIPANIIE--K  521
HGN90406.1_class_I_SAM-de   419  TFLVLYLRRLREYAEEHY-----MVDQLVDYALRNVVGYDLNPLAVLAARTNYLLAIGDLLTHAK-GTVEIPVYLADSILVETKS-DLRG-------QVYVLRAAAGVFEIPKSVVD--G  522
WP_125672489.1_N-6_DNA_me   414  TFLVLAIKRLKEYAEEHY-----LRDITPNYIFKNVVGFDLNPLAVLAARTNYLLVMADMLSYAK-GPVEIPVYLADSLLVEAKS-TL-------KGEMYVIRTYVEEFELP-RSIV-ER  517
RLG60388.1_class_I_SAM-de   414  TFLILAMKRLREYAEEHY-----LRDVLAGYMLKNIVGFDLNPLAVLAARTNYLLAIADLLTYIK-GSIEIPIYLADSLLVEVKS-TL-------TVTSYVIRTYVGEFELP-KSIV-ER  517
NQE06103.1_hypothetical_p   386  TFLVQAIKEVKDYAEEHFV---TDKSELLRKIVGDVVGIDLNPLAVLASRANYVIALGDLIRYIPTGGIEIPVYLADSISVSRKM-TFS------GEWVVYLTTSEGEFSVP-QEVI-NK  493
RZN40535.1_class_I_SAM-de   409  TFLVLAIKEVKDYAEEHFV---TDKSELLRKIVGDVVGIDLNPLAVLASRANYVIALGDLIRYIPREGIEIPVYLADSILVSRKM-KFT------GEWEIYLTTSVGEFSVP-HDVI-DK  516
PXF53089.1_SAM-dependent_   406  TFLVMVINRIKEYAEEHYE---IDKRTLITKIVNNVQGFDLNPLAVLASKANYLIAMAEFLRYRPKEGIEIPVYLADSIAVSRRM-EPT------GQWAVYMKTSEGEFSVH-HEVI-DK  513
TkoII                       399  TFLVLAIKRAKEWGLRHWGTSADAQLRLLESITSNIVGIDLNPLAVLAARGNYLIALGKLIRYRV-DDITIPVYLADSITVSTKT-SAR------GGKRIALKTVAGEFEVP-KEVI-DS  508
HDJ25919.1_class_I_SAM-de   424  TFLVLAIERLKEVVEENWELYAGRLDFVIKSITENVVGFDLNPLAVIAARANYLIQLGRTLLWARREPIEIPIYLADSILIERKP-QL-------TGDVLVLSTSVGDFEVP-AEVI-EK  533
TsoI______TARCCA__1116_aa   429  TFPVLVIGRMLELGRLLM----VPERDLLEAILKNVVGFDLNPLAVLTARVNYLLAISDLLQYRQ-GDITIPIYLADSVRTPAEGQDLF------SQGIFVFPTAVGDFQVP-AVLVTAP  536
TspDTI_____ATGAA__1112_aa   429  TFPVLVIGRMLELGRLLM----VPERDLLEAILKNVVGFDLNPLAVLTARVNYLLAISDLLQYRQ-GDITIPIYLADSVRTPAEGQDLF------SQGIFVFPTAVGDFQVP-AALVTAP  536
TthHB27I__CAARCA___AAS822   441  TFLVLAIRALKVNCFLAG----FSEADTLEVILNSVVGIDLNPLAVTAARVNYLLAIADLLPYRR-REVEIPVYLADSILTPARGEGLF------AQNRRILETAVGPLPVP-EVIN-SR  547
Tth111II__CAARCA__AAU2150   426  TFPVLAIRALKVNCFLAG----FSEADTLEVILNSVVGIDLNPLAVTAARVNYLLAIADLLPYRR-REVEIPVYLADSILTPARGEGLF------AQNRRILETAVGPLPVP-EVIN-SR  532
HauII_____TGGCCA__1093_aa   399  NFLIHAIERYKQAAHAQG----WDDSAILHGITNHIFGFDLNPLAMLTARVNYLIAISDLLKTSS--AVEIPVYQADAVYIPKPR-PDDPTIYRYDISTRLTGCPILALDIP-VSLIHKQ  510
Consensus_aa:                    TFLlhhIpch+phtcbh.......pp.lhp.lhpslVGhDLNPLAVLhARhNYLlhltDLl.h....slEIPlYLADSlhl..c...h...........hh.oshG.hplP...l....
Consensus_ss:                    hhhhhhhhhhhhhhhh       hhhhhhhhhh eeeee   hhhhhhhhhhhhhhhhh                                                     hh   h

Conservation:                     5    5         5          6                            67  5  96   567 59 55 79 988        7 89676999997795 99   88  5  68  957
NAZ27795.1_class_I_SAM-de   527  DLLRKVLDEVRTCLENKCNPSDFAQRLKP-YN-----IDPTDADIILDLYNKLLDLERQGKDKVWVSILRNAFAPILK---GKFDYVIGNPPWVNWESLPEQYREVSKSLWEKYGLA-EI  636
NAZ33036.1_N-6_DNA_methyl   528  DLLRKVLDEVRTCLENMCSSSDFSQRLKLYT------LDPAEVEILLDLYNKLLDLERQGKDKVWVSILRNAFAPILK---GKFDYVVGNPPWVNWENLPEQYREISKGLWIKYGLT-RT  637
AFA38723.1_N-6_DNA_Methyl   526  GLLPDVLAEVAQALRNRYTHKEFSERVKYRFRE----LGEEDVEMLAQLYEKLYELEKEGKDAVWIAVLRNAFAPVLK---GKFDYVIGNPPWVNWENLPETYREISKGLWVRYGLA-EI  637
WP_011007124.1_class_I_SA   526  GLLPEVLSEVAQALRNRYTPKDFAERVRYRFKE----LDQGDVNILKGLYEKLYKLEEEGKDAVWISVLRNAFAPVLK---GKFDYVVGNPPWVNWENLPESYRNVSRDLWAKYGLA-EI  637
Nar7I______CTGRAG__1128_a   530  DLLRKILDEIKMCLENGCTPKEFEFRISPYG------LNSSEKKSLIDLYKKLLDLRLQGKDDVWIAIIRNAFAPILK---GKFDYVIGNPPWIRWDDLPEQYRESSKELWKKYGLL-EI  639
WP_012608870.1_N-6_DNA_me   526  GLLPRVLGEIVGGLNSNYSVKDFKNRVKHEFRD----LSDAEVEVLGKLYETLLKLEKEGKNDVWVSIIRNAFAPMLK---GKFDYVVGNPPWVNWESLPEAYREASKILWSQYGLA-EI  637
WP_012186110.1_N-6_DNA_me   522  NLLPDILHEVTDALRNRYKPEEFKARLQYRFKE----LGENELSVLLDFYSTLLKLEEEGKDDVWVSIIRNAFAPILR---GKFDYIVGNPPWVNWENLPEDFRELSNDLWQHYGLA-EI  633
HGN90406.1_class_I_SAM-de   523  GFLSNVLNDVKMAVANRYTVGEFKGFVGSRYG-----LSDVDVDVLGKLFDVFLRLEKEGKNHVWVSIVRNAFAPILK---GRFDYVVGNPPWVNWENLPENYREISRSVWDEYGLT-KV  633
WP_125672489.1_N-6_DNA_me   518  GLLNKLLEAIDRYVRLKYKPEEFKQIAEREIG-----LSGEELDDVKELYGKFLRLEEEKKNHVWVSVIRNAFAPLVLTSQEKFDYVIGNPPWINWENLPEFYRNSTKQLWDDYGLL-KR  631
RLG60388.1_class_I_SAM-de   518  RLVSRLLEIIDRYVRLRYRVEDFKQVVKAELD-----LTDSELKLAGDLYERFLKLEEEGKDHVWTSIIKNAFAPLTI---GKFDYVIGNPPWINWENLPDEYRERTRDVWENYGLI-AR  628
NQE06103.1_hypothetical_p   494  NLLSNVLGVIETCIKGDYSEKEFEKLIEKDFVD----LKEETIASLEELYEKIKELEKEGKNRIWTRLLKNSFAPLLM---GKFDFVVGNPPWVRWGYLPEGYRDATLKLWKDYGLF-SL  605
RZN40535.1_class_I_SAM-de   517  NLLPNVLGVIETCVRGDYSESEFEKLIEKDIGG----LKEDSIATLTELYAKIKNLEKEGKNRIWTRLLKNSFAPLLM---GKFDFVVGNPPWVNWESLPEHYREDTKKLWDYYGLLEKT  629
PXF53089.1_SAM-dependent_   514  GLLSRVLETIEDCVKRGDYSDRDFKNLIKREFE----LKEESIDSLLELYEKILDLERKGKNKIWTSLLKNSFAPLLA---GKFDYVVGNPPWINWENLPVYYRQQTRRLWDWYGLL-KK  625
TkoII                       509  KLMDKVFQAIEDGLRRNMKKGQFIVYLHMKLGGK--RLSDDTIDAISVLYEKLWKLEQEGKDRIWTRVLRNAFAPLTI---GNFDFVIGNPPWINWESLPENYRELTKPLWEYYGLE-KG  622
HDJ25919.1_class_I_SAM-de   534  EKLGRALSIMDECLHRGYTPEQLKAWLKRELRE----LSDNAIEGLAKLYDRFLELEREQKNRIWLRILKNSFAPLFK---GRFDFVVGNPPWVNWESLPEGYRRRTRVLWAHYNLL-PT  645
TsoI______TARCCA__1116_aa   537  KRFDRFCEILESSIRSEVDPQAFLERTRRELDLNPSEWDDNARKLAEELYTKLLDLHRRGLNGLWARLLKNNFAPLTV---GQFDYIVGNPPWVNWEHLPDNYRRSIAPLWARYEIF-SH  652
TspDTI_____ATGAA__1112_aa   537  KRFDRFCEILESSIRSEVDPQAFLERTRRELDLNPSEWDDNARKLAEELYTKLLDLHRRGLNGLWARLLKNNFAPLTV---GRFDYIVGNPPWINWEHLPDEYRESIKHLWLRYRIAGSY  653
TthHB27I__CAARCA___AAS822   548  AKMERLTDLLEEYVRGDFSTEAFLARAKKEIPDL--ADALHADEVLTELYERLRDLHRQGLDGIWARVLKNAFMPLFL---EPFDYVVGNPPWINWESLPQAYREQTAELWTCYGLF-VH  661
Tth111II__CAARCA__AAU2150   533  AKMERLTDLLEEYVRGDFSTEAFLARAKKEIPDL--ADALHADEVITGLYERLRDLHRQGLDGIWARVLKNAFMPLFL---EPFDYVVGNPPWINWESLPQAYREQTAELWTCYGLF-VH  646
HauII_____TGGCCA__1093_aa   511  HLFARVLEEMEESINEHQHTSAFITHLKRNPEFCRVEDHLDWIPYLEKMFNDIQFLESRSWNRIWCRIARNYFASVAI---GSCDIVAGNPPWVRWSELPQLYAEKIKPICDEYGIF-SD  626
Consensus_aa:                    .bh.clhp.lcptlp.php.p.Fb..hc.pb.......p.p.....l.cLYpchbcLcpptbs.lWhpll+NsFAPlhb...GpFD@VlGNPPWlpW-pLPp.YRc.....lW..YGlh...
Consensus_ss:                    hhhhhhhhhhhhhhhh   hhhhhhhhhhhhh               hhhhhhhhhhhhhhhhh    hhhhhhhhhhh hhh          eeeee            hhhhhhhhhhhh

Conservation:                     5     8   7 8 5    86 5    9   76 68 5596 775   8    68 899 6                        6  57957 6 89868 7855  5   8    6
NAZ27795.1_class_I_SAM-de   637  KG-KTGLGKVKKDLSM--LFLARSFDRYLREGGELGFLITFTVFKTQ-AGASFRSFLARNT-----------RIHVIHDLVTLMPFEGATNRTGAVVVEKVCELDRIREGKCRVIDEVRG  741
NAZ33036.1_N-6_DNA_methyl   638  ----ISTGGFKRDLAM--LFLARSFDRYLREGGKLAFLITFTVFKTQ-AGAGFRSFLAKNA-----------KIHVIHDLVTLMPFEGATNRTGAIVIEKVCDLDKINDGKCRAIAKAQN  739
AFA38723.1_N-6_DNA_Methyl   638  KG-KAGLGKVKRDLAM--LFLARCFDLFLKEGGKLGFLLPFTTFKVQ-AGAGFRDFLAKKT-----------RIHVIHDLVTLYPFEGAVNRTAAIVVEKVCSLSDINNDRCPQMGEVYK  742
WP_011007124.1_class_I_SA   638  KG-KTGLGKVKRDLAM--LFVARSFDLYLREGGKLGFLVPFTVFKMQ-AGAGFRDFLARKT-----------RIHVIHDLVTLFPFEGATNRTAAIVIEKVCSLSDTG-GKCTQMIETFK  741
Nar7I______CTGRAG__1128_a   640  EG-KTGINKFKKDLSM--LFLARCFDLYLKVGGKLGFVITFTVFKTQ-AGAGFRNFLARKT-----------KIHVIHDLVTLMPFEGAINRTGIIIVEKVCELDNMNDNECTIIKEIQK  744
WP_012608870.1_N-6_DNA_me   638  RG-KTGLGKVKRDLAM--LFLARCFDLYLKPGGRLGFLMPFTVFKTQ-AGAGFRRFLATKS-----------RVVEIHDMVTLYPFEGAVNRTSAVVVEKFCELKDIGSEKCPQASVVLK  742
WP_012186110.1_N-6_DNA_me   634  RG-KMGLGKVKRDLAM--LFMARCFDLYLKPGGKHAFLMPFTVFKTQ-AGSGFRRFLATKT-----------RVHVVHDMVTLYPFEGAVNRVSAIVVEK-----------PETGNATG  726
HGN90406.1_class_I_SAM-de   634  RG--MGLGKVKRDMAM--LFLVRCVDLYLRDGGVIGFLVPFTLFKTQ-AGAGFREWISSWN------------IRVIHDLVTLYPFEGAVNRTAAIVVCK-------------------  716
WP_125672489.1_N-6_DNA_me   632  TK-GAALGKVKKDIAM--LFAARCLDRFTKDGGRLSFLVPFTVYKTQ-AGAGFREFLASG-TMRGKI-RCPCKVLKIHDLVTLYPFEGAVNRTSLVVIEKSEGT---------------  729
RLG60388.1_class_I_SAM-de   629  RRGRASLGKAKRDMAM--LFTYTCIDRYLKDGGLFGFLITQTVFKSM-AGEGFREFRIPSSSDDPSK-RVPFKVIKVHDLVELLPFEGAQNRTAAIICVKGEET---------------  728
NQE06103.1_hypothetical_p   606  SGMDARLGAGEKDLSM--LFTYVAVDRYLKNGEKLAFLITQEVFKAKGAGEGFRKFKVKNT---------HVKVVKAHDLVSVKPFESAANKTALIILKKGEKT---------------  698
RZN40535.1_class_I_SAM-de   630  KGM----GLGKVRLLIHPLFTARCIDRYLKTGGKFSFLIPFTTYKTQ-AGAGFRKFLVKG-YWKDKKANSPCKVLKIHDLVTLYPFEGAINRTSLIVIEKKGET---------------  727
PXF53089.1_SAM-dependent_   626  TK-GMGLGKVKRDMAM--LFLARCLDRYTKEEGKLAFLIPFTVYKTQ-AGAGFREFLAKG-YWKDEKMKCSCNVEKIHDLVTLYPFEGAVNRASLIIAEKSCKT---------------  724
TkoII                       623  SG--KGMGRVKRDMSM--LFVARTFDRYLKPGGRLAFLVPFTLFKTQ-AGAGFRRFLTYG-RRREPR--IPVKLLKVHDLVTLYPFEGATTRTGLLVIEKTEKT---------------  718
HDJ25919.1_class_I_SAM-de   646  GR---GLGRVKKDIAM--LFVYRCMDLYLREGGMFGFLITQTVFKSM-AGTGFRNFTIPS-MTRDKT--TPFRVLKVHDMVEFKPFEGAQNRTAIIICRLGEPT---------------  740
TsoI______TARCCA__1116_aa   653  KGFDAILGKSKDDISV--LMTYTVMDKLLKDKGRLGFVITQSVFKTGGGGQGFRRFRIPQG--KEQF--TPLAVIHVDDMVDLNPFEGASNRTAVMVLEKGKPT---------------  750
TspDTI_____ATGAA__1112_aa   654  TGGRPRLGAVKVDISA--LMTYIVVDNLLKNGGKLGFVITQSLLKTA-AGAGFRRLSIPTE--KGKE--VPLRIVYVDDMVDLNPFEGASNRTAVIVLEKGKPT---------------  750
TthHB27I__CAARCA___AAS822   662  SGMDTILGKGKKDAST--LMTYAVADRFLKEGGKLGFLITQSVWKTG-AGQGFRRFRIGENG-------PHLRVLHVDDLSSLQVFEGASTRTSAFVLQKGRPT---------------  755
Tth111II__CAARCA__AAU2150   647  SGMDTILGKGKKDAST--LMTYAVADRFLKEGGKLGFLITQSVWKTG-AGQGFRRFRIGENG-------PHLRVLHVDDLSSLQVFEGASTRTSAFVLQKGRPP---------------  740
HauII_____TGGCCA__1093_aa   627  E---RFFGGNELDISG--MITYTVIDQWLDEGGKLAFILPQNHLQSQ-SSGGFRQFQIRET---------PLEVLQVDDFSEVKPFRRVGNRPAVITIYKGRAT---------------  715
Consensus_aa:                    p.....hG.sK.Dhth..Lhhh.hhD.@L+pGG+htFllsboh@Kob.AG.GFRpF.h..............clh.lcDhsplbPFEGA.NRhthlllbKs..h...............
Consensus_ss:                                hhhhh  hhhhhhhhhh      eeeeee   hhh      hhhhhhhhhh                  eeeeee                  eeeeeeee

Conservation:                         7    6    9                  6 66    6             9             65     9                5             8  96       6 575  5       555      6
NAZ27795.1_class_I_SAM-de   742  LNMSG---VRNVVWV----GGQIDPDTPLEDVLKATRHYEMVMIPLM---PNDPASP----WMQVTPNITNAVRKIIGESQHYEAHAGVYV-GLNQVYFVRVLGR-TPDGMLIVTNPPES  845
NAZ33036.1_N-6_DNA_methyl   740  ANMGG---VKHVVWV----GRQVDPDTPLGEVRETTKSYELVMVPLT---PNDPASP----WMQVTPNIANAVRKVIGGSRYYEAHAGVYT-SLNQVYYVKVLGR-TPDGMLVITNPPES  843
AFA38723.1_N-6_DNA_Methyl   743  SNIAG---VRHIIWANTS-RRPIPTSMPLEEVLKMARRFEAVMTPII---PNNPASP----WMQATPSTIKLIRKLVSGTQYYEANEGVNV-ALNQVYYIRILDK-LPNGMLRITNPPEP  849
WP_011007124.1_class_I_SA   742  NNING---VRHVIWVNTS-RRAIPTDMPLEEVLKRTRRYEAVMAPMM---PNDPSSP----WMQATPSVIKLIRKLTSGNQYYEAHEGVNT-ALNQVYFVKILEK-LPNGMLKITNPPEP  848
Nar7I______CTGRAG__1128_a   745  ENMNG---IKHIVWT----GKKIDPNTSLKDVIKMTKHYEITMIPLI---LNDPTSP----WMQVKPNIINSIRKIINGNQYYEAYAGVYT-ALNQIYYIKVLEK-TPDGMLIITNPPES  848
WP_012608870.1_N-6_DNA_me   743  ENKK----VEHVIWVNKL-GGPIPTDTPLEEVVRVTDRLDAVMVPVV---EGDASSP----WMQVTERILPYVRKMTRGSSPYKAHEGVNV-ALNQVYYIQVKGK-TPDGKLVVTNPPEP  848
WP_012186110.1_N-6_DNA_me   727  NKGG----VRHIIWVNRT-GKPIPTDAHLEDVLKVTERFEAIMAPVV---ERDPSSP----WMQVTSKVLPYIRKITSGTSPYEAHAGVYT-ALNQVYFIQIKQR-LPDGKLLIINPPEP  832
HGN90406.1_class_I_SAM-de   717  EKRSGPLVFRHVLWRNVS-MRPVPPESSLEEVRRSVERVDCLMAPIE---ANKPSSP----WAQTNEIIMRAMQKIL-GKSEYRAYEGVNT-AFNQIYYVDILDK-TADGLLVVQNPVRG  825
WP_125672489.1_N-6_DNA_me   730  --KFP---VDCVIWHNPR-SGGVDQEAELEEVKRTTKQFNMKFLPIE---KDKPGSP----WMQITDKAYEGIKKVIGSSPWYRAHAGVYT-GLNQVYWIKITSE-LPDGLLITNPPLP-  833
RLG60388.1_class_I_SAM-de   729  --KYP---VPYILWRGPR----IDQDLELDEVLKLTSRIKLDARPL----KSRSG-P----WSTLKPKTYLAIKKVINKNNYYKAYAGVFT-GLNAVYWVKILDK-LSDEYIIVENITK-  827
NQE06103.1_hypothetical_p   699  --EYP---VPYVLWRKKK-GAMMNAGISYKEALDRTERVEMQAKP-----IGTPVSS----WQTFEAKSERILDTLRGTSIY-KAHRGASI-DPYGVFYMKIKQA-FADDTILVENLPE-  799
RZN40535.1_class_I_SAM-de   728  --EFP---IPCVMWNNPR-SRGMDQEANLEEVTEATKQFDMILAP-----IRIGKANVNMPWMIIGERVYDILQRIMRPSEY-KAHAGVCT-WADGIFWINILSK-QP-SGFLVSNVGK-  831
PXF53089.1_SAM-dependent_   725  --EFP---VYCVMWHNPR-SKGIGQEAELEEIKKTTRQFDLAFIPIK---ENKPESP----WMEITEKAYEGIKKVIGGSRWYKAHEGVKT-ALNQVYWVEILSE-EPNGLLITNPSLP-  828
TkoII                       719  --EFP---VPLIVWNWPD-TRGADVEATLEEVKEKAAIHQLVFMPIE---EGKPESP----WMMTTVKAYEGLKKALGSSEY-RAYAGVFT-GLDGAYWVEIIRE-VAPGRVLIQNVGK-  821
HDJ25919.1_class_I_SAM-de   741  --EYP---VPYVLWKKAV-RGEIPTDASLSEVLAMTKRYELEARPLD---PEVRNSP----WATLTGRAYNAVRKLIGRSAY-VAHAGVYC-GLNAAYWVEVLCE-APDGRLLVRNVLE-  843
TsoI______TARCCA__1116_aa   751  --KYP---VPYTVWRKKR-GTRFTYDSTLDEVLSATRRLHFFAEPVD---PKDPTSP----WLTARPKVLKAVRKILGKSDY-RARKGVTP-SVNSVFWLIEALK-RPDGLILIHNLTE-  853
TspDTI_____ATGAA__1112_aa   751  --QYP---VPYTVWRKNR-GVRFTYDSTLEDVQAATRRLQFQAQPVD---PDNPTSP----WLTARPKALRAVRKILGQSDY-EAHAGVYTGGANAVYWVEPVLK-RPDGLWVVRNLTE-  854
TthHB27I__CAARCA___AAS822   756  --RYP---VPYTYWKKTTKGEGLDYDSTLGEVMEQTKRLRFHAVPVD---PDDLTSP----WLTARRRALYAVRKVLGTSEY-RAYEGANSGGANGIYWLEILAE-RPDGLVVVRNVTE-  860
Tth111II__CAARCA__AAU2150   741  --RYP---VPYTYWKKTTKGEGLDYDSTLGEVMEQTKRLRFHAVPVD---PDDLTSP----WLTARRRALYSVRKVLGTSEY-RAYEGANSGGANGIYWLEILAE-RPDGLVVVRNVTE-  845
HauII_____TGGCCA__1093_aa   716  --TYP---VPYNIWKRIT------PTTISENVSYATAAENLLSIPHEAYALEDAGKR----WSILPLGRFPLLSMLNGEDRSIEGRKGIVT-DLNGAYFIRILGHGYRTGTLRFKTSPD-  818
Consensus_aa:                    ....s...V.hhlW.p.p....hs.ps.Lc-V...Tpphph.h.Pl.....ppssSs....W..hp.phh..lp+lhs.sp..cA..Gl.h.shN.hY@lclh.c..ssG.llhps.sc.
Consensus_ss:                            eeeeee                hhhhhhh  h  hhh                       hh   hhhhhhhhhh          ee              eeeeehh                h
```

```
Conservation:                     8  6  6  57 575796589 575 5855      65 6                   56    77   5   8  6
NAZ27795.1_class_I_SAM-de  846 GAKKRVRQVEARVEPDLVYPLIRG-EDVGRWYVDYKDRYVIIPHNP--RTASPIPEGDMRVRWPLTYSYLVNYRRELEDRS---------------------------------------  923
NAZ33036.1_N-6_DNA_methyl  844 GQKKEVKQVEAKVEPDLVYPLIRG-EDVKKWYVEFKDRYVIVPHNP--RTGQPYRLNEMITRFFNAFSYLNEYKDELKKRSIK------PFLSLR-------------------------  929
AFA38723.1_N-6_DNA_Methyl  850 GQKKTVKQVEAIVEPDLVYPLIRG-RDVRRWYVGFEDRYIIVPHDP--KTGKPLRESDFKVKFPQTYDYLNTYKRELEGRS---------------------------------------  927
WP_011007124.1_class_I_SA  849 GQKKKVKQVDAVVEPDLIYPLIRG-RDVKKWYVGFEDRYIILPID---SRGDPISHDKMKTKYPNTYDYFFRFFKELISRSGE------PYKSKL-----------------------EP  935
Nar7I______CTGRAG__1128_a  849 GQKKNVKQVEARVESDLIYPLVRG-RDIKKWYVEFKDKYIILPHD---KEGDPIPHHIMKTKYPYTYQYFYQFFNDLINRSGG------FLKQKM-----------------------AP  935
WP_012608870.1_N-6_DNA_me  849 GQKKKVEQVEAVVEPDLVYPLIRG-RDVKKWYVEYKNRYIIVPHDP--KTGDPISHFDMKVRYPLTWKYFHNYFNDLISRGGE------PYKSQL-----------------------KP  936
WP_012186110.1_N-6_DNA_me  833 GQKKRVKQVEVTIEPDLVYPLIRG-RDVKRWYVDYKERYIILPTD---EQGNMIKHYEMKIKYPNTYRYFYEFFRDLVNRGGE------PYKSKL-----------------------EP  919
HGN90406.1_class_I_SAM-de  826 GEKKTVEVVTAKVEPELVYPLIRG-RDIKKWYVKHENRYIILPVK---EDGENFHHKDLRTKYPHVWQYFRNFFDELISRGGE------PYKTKL-----------------------KP  912
WP_125672489.1_N-6_DNA_me  834 GQKKTVKQVEQIVEKELVYPLIRG-RDVKRWYVAGDYGWIIIPHDP--ETGDPIKESEIKINFPKAYGYFSNFKKELESRSIH------KLWGK--------------------------  918
RLG60388.1_class_I_SAM-de  828 GAKRKVKQIRSPIDKKFLYPLLRG-RDVKKWLAKPKF-YIILPTD---NNGKNISINNMRINFSKTYEYFLNFLDELITRGGQPYKDNLKPWREK-----------------------PK  919
NQE06103.1_hypothetical_p  800 LGKRKISKIEERIESNLVFPAVRG-RDIKRWKAEPE-IYALIVQDP--KKRMGYEEEWMKVNLPRTFAYLHKFKELLLSRQSY-----WKYFSKEVLDSKDSSEKLKELHRYVRSVGKRI  910
RZN40535.1_class_I_SAM-de  832 TAKATTKKITQLVDSEFIFPVLRG-KNLKKWYARSE-GHILLPISS--NGEILTDEE-LKINYPNTYSYFFDFSKELRRRSGY----------KQ-----------------------LL  913
PXF53089.1 SAM-dependent   829 GQKKKVKQVKKVVEKDLVYPLVRG-RDVKKWYVKGDLGYILMPVNG---NGKTLSHSDLKVKYPETYQYLSNFVGDLVNRGGE------PYKTKL-----------------------KP  915
TkoII                      822 TMKKSIKRVQKVVETDLIFPVLRG-KNAKKWYCNPE-GWILLPIDK---NGDIIPPSELKLKYPLTYDFFTALLDELISRNAE------PFKTKL-----------------------RI  907
HDJ25919.1_class_I_SAM-de  844 RAKKKVRQVEWAVEPELVYPLLRG-RDVARWLARPT-LYIVVPTDK---HGINLPENELKVRYPGVFGFFTNFKEELKSRSIY------KLAGKG-------------------------  927
TsoI______TARCCA__1116_aa  854 GAKVEVGEVSETIEPDLLYPLLRG-RDVKRWRAEPS-AMILITHEP-GMRLKAIPEKEMQTRYPRTYGYLKRFEQVLRRSAVF----------RR-----------------------YF  937
TspDTI_____ATGAA__1112_aa  855 GAKVKVEEVTETIEPDLLYPLLRG-RDVQRWRAEPS-AWIIIPQDP-NNPSRAYPEAKLKVDYPRLYAYLKQFESVLRRRAAY----------QQ-----------------------IL  938
TthHB27I__CAARCA___AAS822  861 GAKREVEGITTELEPDLLYPLLRG-RDVRRWYAQPS-LHILMVQDP--KTRRGIDEQVLQKRYPKTWAYLKRFEAVLRERSGF----------RR-----------------------YF  943
Tth111II__CAARCA__AAU2150  846 GAKREVEGITTELEPDLLYPLLRG-RDVRRWYAQPS-LHILMVQDP--KTRRGIDEQVLQKRYPKTWAYLKRFEAVLRERSGF----------RR-----------------------YF  928
HauII_____TGGCCA__1093_aa  819 QGQKPVPERTYEIEADLVYPLIKGAKNVQPFYATTSELAVIVPNKGINSSSMPSMSRLTYQGYPLAARYFQSLNRDVLIDGVGLLDQRSTWRTRM-----------------------RP  915
Consensus aa:                  ..K+pVpplp..lEs-LlYPLlRG.+slp+Whhp.p..@llls.c...pp..sh.pp.hp.p@P.h@.Yh.p@.p.L.pRts..........+...........................
Consensus ss:                  hhhhhhhhh     hhheee                  eeeee            hhhhhhhhhhhhhhhhhhhhhhhh

Conservation:                                6          88    6   55 6798 9   7             6                   568       5     69575 55899        57
NAZ27795.1_class_I_SAM-de  924 -----IHKLWGRSNP----------FYAVYDIGTYTFAPYKVVWGAIAGAITGKAVSFACAVIE------PIN-----GRPVMPDHSVILVPTETSDKAYYIAGVLNSVVVRSIIASYTY 1017
NAZ33036.1_N-6_DNA_methyl  930 ---DKIKKAKSQANKERTEEELNKNFYILDNIGAYTFTPYKVVWKRIAGAITGKAVSFACAVVE------PVD-----GKPVIPDDGTILVEAKTPDEAYYIAGVLNSIVARSIIASYTY 1035
AFA38723.1_N-6_DNA_Methyl  928 -----IHKLWGKGNP----------FYSVYDIGTYTFAPYKVVWKRIAGAITGKAVDFACVVVD------PITNRFFTSKLVIPDDSTIMVGFNNSDEAYYVAGILNSTPVRAAIASYTY 1026
WP_011007124.1_class_I_SA  936 YRKLPFEKAEKIAPP----------FYWIFNV-VPSLAPYKVIWKSIAGAITGKAVDFACAVVG------PFTDKFLGSKIAIPEHSTIFIGFDNADEAYYVAGVLNSTPVKTVIASYTY 1038
Nar7I______CTGRAG__1128_a  936 YKKYPFYKAEKMTVP----------FYYVMNNVKASLAPYKVVWTRIAGAITGKAVSFACAVVE------PIN-----EKPVIPDDGIILVEAKSPNEAYYIAGVLNSTIVRSIIASYTY 1034
WP_012608870.1_N-6_DNA_me  937 YRELGLEKGGQGAPP----------FYYLFNV-KHAFAPYKVVWKEVSARM--KAGGFHVAVVG------LVEDEHLGSRVVVPNHTVVMIPAQDEEEAYYLAGVLNST-----FTQFAL 1032
WP_012186110.1_N-6_DNA_me  920 YRRLPLEVAEKSAPP----------FYWVFNV-EPSLVPYKVVWSRIAGGISGKAVSFSCAVVE------PIEIKDLGKKPVIPDDGTILIDLKDPKEAYYIAGVLNSIIVRTTIAAYTY 1022
HGN90406.1_class_I_SAM-de  913 YRIKDIHVAEKSAPP----------FYWVFNA-APSLATYKVVWKEVSARM--KAGGFHVALLE------PHD-----NIPVIPDHTAVLIPCETKDEAYYLSAILNSTVV-GLYAAYI- 1006
WP_125672489.1_N-6_DNA_me  919 ------------GNP----------FYSVYDIGDYTFYPYKVVWKRIAGAITGKAISFASAVLEP-VKAEFLG-----NRPVILNDSLILIPFKDEEEAYYVTGVLNSSIALLTIASYTY 1010
RLG60388.1_class_I_SAM-de  920 EIAEKI------APP----------FYMLFNVKQ-SFALYKVLWKE-------QAKGLIAAVIDS-INDPILG-----IKIVIPNHKLMFVPCEKENEAHFICAVLNSIIARLIAKSYII 1009
NQE06103.1_hypothetical_p  911 RNGKELYVFQVSNAP----------FYTMFNIGEQSFAPYKVVWKRMASDLEAV--------VLS-KVKTPIG-----EEDVIPTDTTSLIPFKSEEEAHYVCAILNSLPVRFCVRSYSS 1006
RZN40535.1_class_I_SAM-de  914 EK---------SNKP----------FYSVLR-SKYAIMPYKVAWKHISGKISGK--------ALF-ECSVIS----EEEKTIIPTHGVINIACESEDEAHYICSILNSSFASLIVMSYSL 1000
PXF53089.1_SAM-dependent_  916 YREKPFEDAERVAPP----------FYWLFN-AEPSLAPYKLVWKVIAGGITGKAVRFASSVLEP-IKIGYIEK----ESPIIPDASLILISFNDLEEAYYVSAVLNSSIDLLAIASYTY 1019
TkoII                      908 YKEKPIEIAERSGMP----------FYAVLN-SQVSIAPYKVGWKHISGAISGK------AQLDV-FVIPTLQ-----DKPMIPTHGIMHIPTSSEDEAHYIASILNSTIARFIVASYGL 1004
HDJ25919.1_class_I_SAM-de  928 ------------GPW----------YGLYVDIGPYTFAPYKVVWREQAAELTCA-------LVPP-VEDRYVG-----KKVVIPAHKLMLVPLKDEDEAHYLCALLNSSPVRLLVKSFMV 1012
TsoI______TARCCA__1116_aa  938 IRKKGGEL--VDAGP----------FYSMFNVGDYTFAPWKVVWREIASDLTAA-------VVS------SRE-----GKTIVPDHKLVLVACSSDIEAHFICALLNSSIVRFVALGYAI 1027
TspDTI_____ATGAA__1112_aa  939 CKREPE-------------------FYGIMDIGHYSFSPWKVVWTRLAK-IEAA-------VVG------LHG-----RKPVIPQETVSLVQCDTKEEAYYIAALVNSTAFQLAATSYSQ 1020
TthHB27I__CAARCA___AAS822  944 TRKDRNGRM-VETGP----------FYSMFNVGDYTFAPWKVVWRYVASDFIVA-------VVG-----PASD-----EKPVVPNEKLMLVPVEDDNEAFYLCGVLNSSPIRFAVQSFFV 1035
Tth111II__CAARCA__AAU2150  929 TRKDRNGRM-VETGP----------FYSMFNVGDYTFAPWKVVWRYVASDFIVA-------VVG-----PASD-----EKPVVPNEKLMLVPVEDDNEAFYLCGVLNSSPIRFAVQSFFV 1020
HauII_____TGGCCA__1093_aa  916 FLEKQY-ADNLADIP----------FYAIYNVGDYTFAPYKVVWAEMSGSLKAA-------VISDGLVPYIEQ-----RKIIIPDHKIYFASFSSEKYAHFICALLNSSIIREFVDSFTI 1012
Consensus aa:                  .....b......s.P..........FY.hhsltp.ohtPYKVVWpphtt.lpt........Vl...............p+.llPsc.hhhlshps.pEA@@lttlLNSs.hp.hh.S@.h
Consensus ss:                                                           eeee      eee       ee                  eee   eeeee   hhhhhhhhhhh  hhhhhhhhhh

Conservation:                  5         5     68 7   5  5  7 569  99  6                               7                 6   79 5   8 8 8     68 55   5
NAZ27795.1_class_I_SAM-de 1018 E---LGQYTHIIDVIKIPKFDPSNDLHRRIAELSRRAHELARCIHA--GSK--------PSYCAGV-NAGDELR--------------RVEGELDSAV---AKLYGLS-EGELKEFRRLM 1105
NAZ33036.1_N-6_DNA_methyl 1036 E---LRQETHIVDVVRIPKFSPNNDLHRRIAELSKRAHELARCVYA--SNK--------PNYCADI-NAEDELR--------------RVEDELDLAV---ARLYGIP-EGELKEFRRLM 1123
AFA38723.1_N-6_DNA_Methyl 1027 E---LRQETHIVDVIKIPRYDSKNELHRNISELSKRAHELAREIYG--KGR---------------RDLEGELR--------------KVEEEIDKAV---AELYGMT-SEELKEFERLL 1108
WP_011007124.1_class_I_SA 1039 E---LRQETHIVDNVKIPKYDPENELHKKIAELSKRAHDLAKEIHE--ENR---------------RNLETELR--------------KVEEELDIVV---AKLFGMG-EAELREFKKLL 1120
Nar7I______CTGRAG__1128_a 1035 E---LRQETHIIDIIKIPKFDPDNNLHNKITELSERAHELAKCIYS--NNK--------PDYCKNI-NAKEELK--------------KVEEKIDLAV---AELYGIS-KEELEGFKELM 1122
WP_012608870.1_N-6_DNA_me 1033 Q---YAVVASVEEYISVPKFNSKSELHIKIAEISRKAHEIAKCLHS--QVK--------PDYCKNIRDPEGELA--------------RVEDELDKAV---AQLYGIP-EEAIEDFRKLL 1121
WP_012186110.1_N-6_DNA_me 1023 E---LRQETHIVDFIRIPRYDPNNELHGRISELSRRAHEIARCIHA--SAK--------PEYCGFIRDPEGGLR--------------KIEDELDRAV---AQLYGIP-EDALEEFRKLL 1111
HGN90406.1_class_I_SAM-de 1007 -------TVKGIETLRIPQFDPRNPMCKKLIEFSAKAHEIAKQIYE--HND---------------EHRKKELA--------------EIEEEIDKLV---AELYGLS-DVELAEIKRFF 1084
WP_125672489.1_N-6_DNA_me 1011 E---LRQETHITQYIKIPEFDSKNSLHERLSQLSRKAHEIAKKIYE--ENR---------------EDLKEDLS--------------KIEEEIDKTV---AQLHGIT-DEELDEIRKCL 1092
RLG60388.1_class_I_SAM-de 1010 E---TQISTHIMDYVNVPQFDDNNDIHRKLAELSKIAHRLVAS-GE--ESK---------------------LS--------------EIEEEIDRLV---AQLYGIS-DDELRDIKISL 1084
NQE06103.1_hypothetical_p 1007 AG-RGFGAPSIVKHFGIPKYERSNEVYRMLSELSKRAHGLAKRYCE--LKD---------------LQAQEELK--------------EVEVGIDKVV---AELYGIT-DEELEEVRETL 1090
RZN40535.1_class_I_SAM-de 1001 E---VHLSYDILQNVYVPKFNSRDKIHLKLAELSKKAHALAKRYYE--QDD---------------LEAQGGLK--------------EVGEEIDRIV---AGLYGIT-DEELEEVRKTL 1082
PXF53089.1_SAM-dependent_ 1020 E---LRQETHITQYIKIPKYNSNNQLHRELGELSKKAHGIAREIYE--EER---------------EDLRDDLA--------------TVEEEIDRKV---AELYGIT-EEELKEIKKCL 1101
TkoII                     1005 E---VHITTDVPKRIYIPTFDPSNQLHLELAELSKKAHEIAKEKYELLDRIKSLKKSLKGTRGEERKRIKDEIRELEEELAEVEKKLGEVEKEIDEKV---AELYGIT-WEELEEIERLY 1117
HDJ25919.1_class_I_SAM-de 1013 E---TQISTHVIERVEIDRYDPGNRIHRELVQLSKRAHELVSSGQF------------------------RELE--------------AVEKRIDELI---AELYGLT-RDELEDVRVCL 1087
TsoI______TARCCA__1116_aa 1028 Q---TQFAPHLLDFIRIPRYNPTDPLHRRLSELSQAAHKAAQAGDE------------------------KRLE--------------ALEAEIDREA---AKLWGLT-EAELREIQESL 1102
TspDTI_____ATGAA__1112_aa 1021 EGGKSMGSMHILEHIRIPRYQPTDPVHRRLAELSQAAHEAAQAGDE------------------------KRLE--------------ALETEIDREA---AKLWGLT-EAELREIQESL 1098
TthHB27I__CAARCA___AAS822 1036 Q---TQIAPHVLQKLCIPRYEPNTDHQNRIAHLSRRAHELAPAAYN--GDK----------------AARAELR--------------RVEEEIDRAA---AQLWGLT-EEELAEIRRSL 1116
Tth111II__CAARCA__AAU2150 1021 Q---TQIAPHVLQKLCIPRYEPNTDHQNRIAHLSRRAHELAPAAYN--GDK----------------AARAELR--------------RVEEEIDRAA---AQLWGLT-EEELAEIRRSL 1101
HauII_____TGGCCA__1093_aa 1013 K---LQV-GTIFKHLRLPKFDLENAEHLSLVDLSITAHNTMNKTNG-----------------------SGNIE--------------AFIEQIDIIAVRLLAKFAATITDQLQQ----- 1086
Consensus aa:                  p...h...splhp.l.lP+@psps.hH.pls-LSppAHphhp.......pc..................p.pLp...............lEpcID..h...ApL@Glo.p.ELp-hcc.h
```

```
Conservation:                        8   6
NAZ27795.1_class_I_SAM-de 1106  AILSGGEVPVEDD------VEV-PKSPVVNVLNTLLKPDVESYIEVDVTNPS-GEEV---EFTYELPW---GRGSF----KVTEGKYR---INTPPLKPGKYRGVVRWVWR---GKEYTQ 1201
NAZ33036.1_N-6_DNA_methyl 1124  AILSGEEVPVED------EVEV-PKEPVVNILNTLLKPNVESYIEVDVTNPS-GEEV---EFTYEFPW---GRGSF----RVTEGKFR---INTPPLKPGKYRGVVRWVWR---GKEHSR 1219
AFA38723.1_N-6_DNA_Methyl 1109  AILSSEEVPEEEE-----GVEI-SERPAVTVLNTSLQPGVGSYIEVDVVNPS-GEEI---VFTYELPW---GKGSF----SMVSGKYR---IPTPPLKPGKYSGVLKWRWR---GEEHSV 1205
WP_011007124.1_class_I_SA 1121  AILSGSEYSEEEE-----EVAV-PEKPTVHVLNTSLQPGVESYIEVDVVNPS-GEEL---VFTYELPW---GKGSF----SIITGRYR---IPTPPLKPGRYSGVLKWRWR---GEEHSV 1217
Nar7I______CTGRAG__1128_a 1123  EILSGK------------------------------------------------------------------------------------------------------------------ 1128
WP_012608870.1_N-6_DNA_me 1122  RAFAGEEVSEEV------SEEE-EVSPSVDFVKTDVVAGQADFIEFSISTSGLCDKA---EVVLEAPW---GSQRL----TLGDGRHR---VEVEGLAEGVYRVRYSFSCG---DYRKSG 1218
WP_012186110.1_N-6_DNA_me 1112  GVLSGESVPEEEE-----SVVG-VVRPSVEFLKVNVVAGQTDYLEVNIVTAGLCDKA---VLILKWPW---GTQTL----NLDDGRHR---IEVK-VPEGVYEVAYSFKCS---GYEYDG 1208
HGN90406.1_class_I_SAM-de 1085  AILAGEEIPEEQI-----VEDE-EAELSIAFPKTVLTPNQPETITIHITNPD-KTPL---QITLTLPD---NQTKT----IQTNSEEHTEDIPITPLPPGTYKLTYTIKTN---NQTIKQ 1184
WP_125672489.1_N-6_DNA_me 1093  AVLKEGEIPEEEEE----ITEE-PEEIKVDFLNTVVKPNAIGVVEISVINPL-KDKL---VIEVGPPSI-PAKLET----SKEEDKFH---IKIQPLEAGEYEVPYKVITSK-GVVEGK- 1193
RLG60388.1_class_I_SAM-de 1085  KIL-EGEEIEEEEL----AEEE-PREVKVDFLEAVVRPNRIGSFEVAILNPL-KERV---TIELQLPEY-PVKLET----DKEEDRLR---VKVPPLKAGEYEIPYKVITSQ-KTIEGS- 1184
NQE06103.1_hypothetical_p 1091  GVLKG-ENSER------------------------------------------------------------------------------------------------------------- 1100
RZN40535.1_class_I_SAM-de 1083  RVLKG-EDVVELEK----N----------------------------------------------------------------------------------------------------- 1096
PXF53089.1_SAM-dependent_ 1102  RILKEGEIEEEEGEEEEGVVLLKEEKIEIELEPLLIKENESQEMEIKIANHL-KTEIQNAVVKVMLEDKPLFKGEIAGIGSGETGALT---FNSPKLRSGQY--PLKVILEHENGKEEGI 1215
TkoII                     1118  TVLMNAEP---------------------------------------------------------------------------------------------------------------- 1125
HDJ25919.1_class_I_SAM-de 1088  SLLLC-EDEKTDED---------------------------------------------------------------------------------------------------------- 1100
TsoI______TARCCA__1116_aa 1103  RELEG-EVPAAEEE----A----------------------------------------------------------------------------------------------------- 1116
TspDTI_____ATGAA__1112_aa 1099  RELEG-EVPAAEEE----A----------------------------------------------------------------------------------------------------- 1112
TthHB27I__CAARCA___AAS822 1117  EELRG------------------------------------------------------------------------------------------------------------------- 1121
Tth111II__CAARCA__AAU2150 1102  EELRG------------------------------------------------------------------------------------------------------------------- 1106
HauII_____TGGCCA__1093_aa 1087  ---EG-FNLEF------------------------------------------------------------------------------------------------------------- 1093
Consensus_aa:                   ..L.s.E.................................................................................................................
Consensus_ss:                   hhhh

Conservation:
NAZ27795.1_class_I_SAM-de 1202  E-FEVEVYE--QKGPRRPRT--LLDF---------- 1222
NAZ33036.1_N-6_DNA_methyl 1220  E-AEVEVSE--PVGPRRPRT--LLDI---------- 1240
AFA38723.1_N-6_DNA_Methyl 1206  D-IVMEVAQ--PEGPRRRST--LFGSR--------- 1227
WP_011007124.1_class_I_SA 1218  D-IVVEVAQ--PEGPRRRST--LFGSR--------- 1239
Nar7I______CTGRAG__1128_a       ------------------------------------
WP_012608870.1_N-6_DNA_me 1219  E-VEVRASKSSPRSPRRPST--FFTQ---------- 1241
WP_012186110.1_N-6_DNA_me 1209  S-VKVTASSKVPEGPRRSRT--LRLG---------- 1231
HGN90406.1_class_I_SAM-de 1185  DTVDITVTQ-----QRRFRT---------------- 1199
WP_125672489.1_N-6_DNA_me 1194  --FTLYVRE-----EERRRTRDLLSSKIDELLDEET 1222
RLG60388.1_class_I_SAM-de 1185  --FTLYVKE-----EKRHRVREELESKLDELLGERL 1213
NQE06103.1_hypothetical_p       ------------------------------------
RZN40535.1_class_I_SAM-de       ------------------------------------
PXF53089.1_SAM-dependent_ 1216  --RTLFVRA-----AKVKKGKGLFDEELEEMLK--- 1241
TkoII                           ------------------------------------
HDJ25919.1_class_I_SAM-de       ------------------------------------
TsoI______TARCCA__1116_aa       ------------------------------------
TspDTI_____ATGAA__1112_aa       ------------------------------------
TthHB27I__CAARCA___AAS822       ------------------------------------
Tth111II__CAARCA__AAU2150       ------------------------------------
HauII_____TGGCCA__1093_aa       ------------------------------------
Consensus_aa:                   ....................................
Consensus_ss:
```

Supplemental Figure 3. PROMALs protein alignment of TkoII and close homologs. The PROMALs multiple sequence alignment server (http://prodata.swmed.edu/promals/promals.php) was used to align TkoII with close homologs and identify the catalytic motifs PD-ExK (green), FxGxG (cyan) and NPPY (yellow). Residues highlighted in red denote predicted alpha helice structure, while residues highlighted in blue denote predicted beta strand structure.

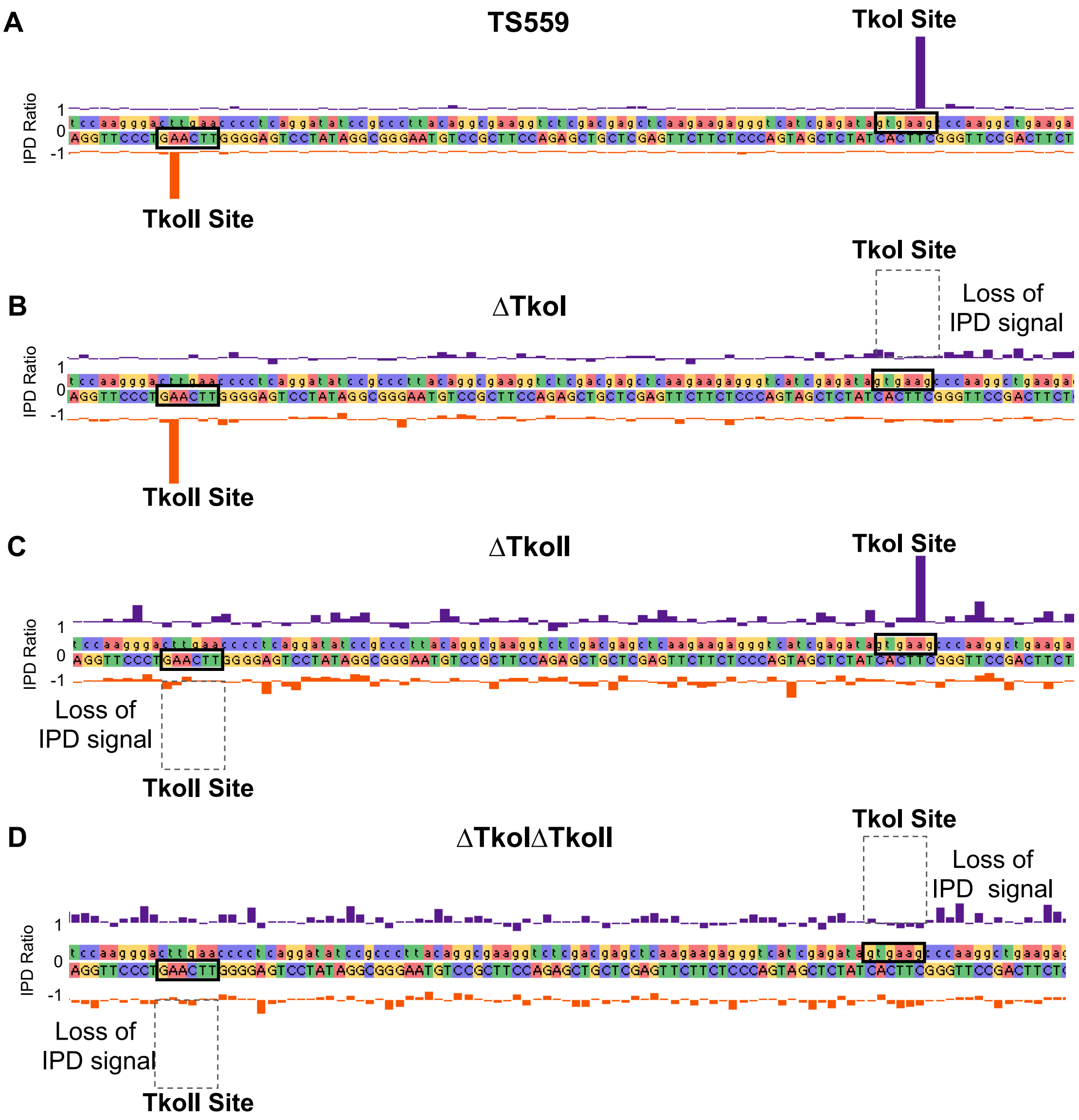

Supplemental Figure 4. Pacific Biosciences SMRT sequencing and IPD ratios of *T. kodakarensis* strains. PacBio SMRT-sequencing can detect the presence of 6mA in DNA by causing a lag in the sequencing polymerase interpulse duration (IPD). PacBio secondary modification and motif analysis provides an IPD ratio (Experimental IPD/Expected IPD) for every sequenced base and outputs methylation motifs. An IPD ratio >3 is indicative of a methylated base. (A) TS559 parental strain shows the presence of two methylation motifs: GTGAAG and TTCAAG, where underlined base denotes location of 6mA. (B) ΔTkoI shows the presence of a single methylation motif (TTCAAG) and loss of secondary motif (GTGAAG), (C) ΔTkoII shows the presence of a single methylation motif (GTGAAG) and loss of secondary motif (TTCAAG) and (D) ΔTkoI ΔTkoII shows loss of both methylation motifs.

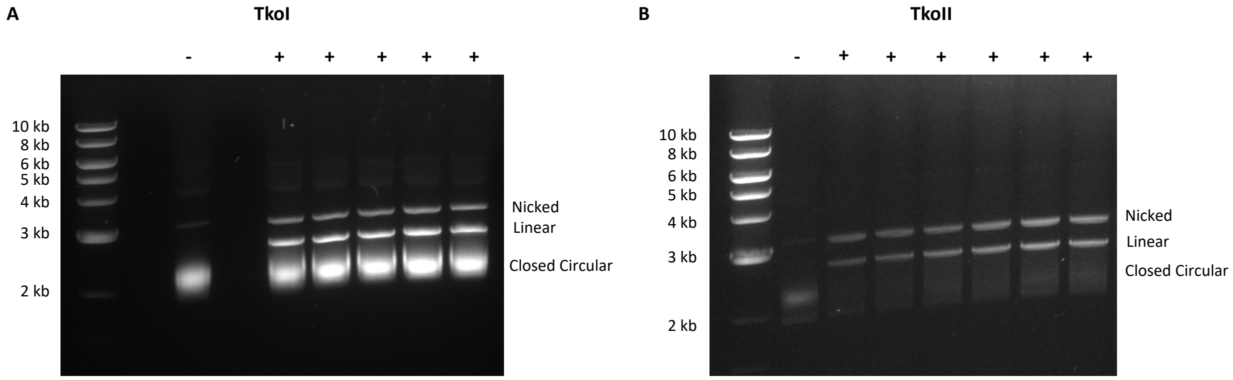

Supplemental Figure 5. REase cleavage activity on pUC19 plasmid. pUC19 (3 ug) plasmid DNA was incubated with (+) or without (-) (A) TkoI (100 nM) in 1X NEBuffer 3 or (B) TkoII (10 nM) in 1X NEBuffer 3 with 80 µM SAM, and 0.2 µM trans DNA for 30 min at 65 °C. Reactions were halted by the addition on 0.5 units of Proteinase K and 10 uL of 6x Purple Loading dye. The linear band was excised and DNA was isolated with the NEB Monarch DNA Gel Extraction Kit.

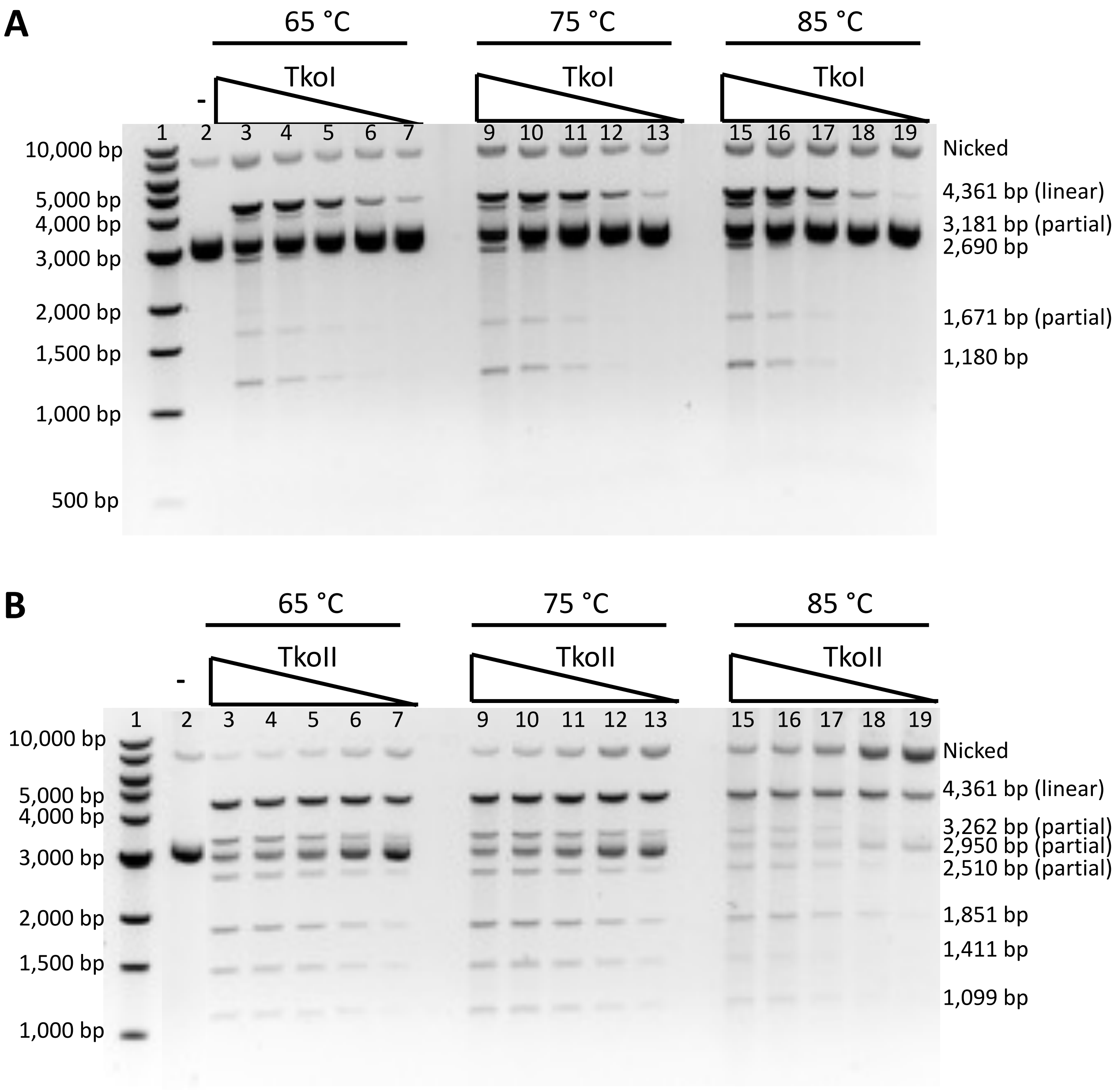

Supplemental Figure 6. Characterization of TkoI and TkoII restriction endonuclease cleavage activities at 65°C, 75°C and 85°C. (A) TkoI was incubated with pBR322 in 1X NEBuffer 3 supplemented with SAM and 90mer *trans*-DNA for 30 min at 65°C (Lanes 3-7), 75°C (Lanes 9-13) or 85°C (Lanes 15-19). Reactions were halted with Proteinase K and loading dye and separated on a 1% agarose gel. Lane 1 is a 1 kb DNA ladder. Lane 2 is pBR322 alone and lanes 3-7, 9-13 and 15-19 are decreasing amounts of TkoI (100 nM – 6.25 nM). (B) TkoII was incubated with pBR322 in 1X NEBuffer 1 supplemented with SAM and 90mer *trans*-DNA for 30 min at at 65°C (Lanes 3-7), 75°C (Lanes 9-13) or 85°C (Lanes 15-19). Reactions were halted with Proteinase K and loading dye and separated on a 1% agarose gel. Lane 1 is a 1 kb DNA ladder. Lane 2 is pBR322 alone and lanes 3-7, 9-13 and 15-19 are decreasing amounts of TkoII (10 nM – 0.62 nM).

1.2 Supplementary Table

Supplemental Table 1. Putative *Thermococcus kodakarensis* defense systems predicted using the PADS (Prokaryotic Antiviral Defense System) Arsenal database (https://bigd.big.ac.cn/padsarsenal/).

Assembly accession	System name	Gene symbol and type	Product accession
GCA_000009965.1	ABI	abiLi | AbiL	BAD84208.1
GCA_000009965.1	BREX	BrxP | type IV	BAD84382.1, BAD85149.1, BAD85504.1, BAD85745.1, BAD86010.1, BAD86356.1
GCA_000009965.1	BREX	BrxHI | type II, type VI	BAD84953.1, BAD85204.1
GCA_000009965.1	CRISPR-CAS	Cas6 | type I-A, type I-B, type I-D, type I-E, type III-A, type III-B	BAD84636.1, BAD84653.1, BAD86442.1
GCA_000009965.1	CRISPR-CAS	Cas2 | type I, type I-B, type I-C, type I-C-V, type I-D, type I-E, type I-F, type I-F-V2, type II, type II-A, type II-B, type II-C, type III, type III-A, type III-B	BAD84634.1
GCA_000009965.1	CRISPR-CAS	Cas4 | type I-A, type I-B, type I-C, type I-C-V, type I-D, type II-B	BAD84258.1, BAD84647.1
GCA_000009965.1	CRISPR-CAS	Cas8a1 | type I-A	BAD84652.1
GCA_000009965.1	CRISPR-CAS	Cas5 | type I-A, type I-B, type I-C, type I-E, type I-F-V2	BAD84641.1, BAD84650.1
GCA_000009965.1	CRISPR-CAS	Cas3" | type I-A, type I-B, type I-C, type I-C-V, type I-D, type I-E, type I-F, type I-F-V2	BAD84638.1

GCA_000009965.1	CRISPR-CAS	Cas7 | type I-A, type I-B, type I-C, type I-C-V, type I-E, type I-F-V2	BAD84642.1, BAD84651.1
GCA_000009965.1	CRISPR-CAS	Cas3' | type I, type I-A, type I-B, type I-C, type I-C-V, type I-D, type I-E, type I-F, type I-F-V2	BAD84639.1
GCA_000009965.1	CRISPR-CAS	Csa3 | type I-A	BAD84637.1
GCA_000009965.1	CRISPR-CAS	Csa5 | type I-A	BAD84643.1
GCA_000009965.1	CRISPR-CAS	Cas1 | type I, type I-A, type I-B, type I-C, type I-C-V, type I-D, type I-E, type I-F, type I-F-V2, type II, type II-A, type II-B, type II-C, type III, type III-A, type III-B	BAD84644.1
GCA_000009965.1	DISARM	DrmC | type I, type II	BAD85752.1
GCA_000009965.1	DISARM	DrmB | type I, type II	BAD84710.1
GCA_000009965.1	DISARM	DrmA | type I, type II	BAD84495.1, BAD84639.1, BAD84649.1, BAD84710.1, BAD85117.1, BAD85204.1, BAD85210.1
GCA_000009965.1	DND	DndC | ------	BAD85206.1
GCA_000009965.1	DND	DndH | ------	BAD84276.1, BAD84790.1, BAD85545.1
GCA_000009965.1	DRUANTIA	DruE | type I, type II, type III	BAD84495.1, BAD84639.1, BAD84649.1, BAD84659.1, BAD84710.1, BAD84755.1, BAD85117.1,

			BAD85204.1, BAD85210.1
GCA_000009965.1	GABIJA	GajA | ------	BAD84962.1, BAD85584.1
GCA_000009965.1	HACHIMAN	HamB | ------	BAD84495.1, BAD84639.1, BAD84649.1, BAD84659.1, BAD84710.1, BAD84755.1, BAD85117.1, BAD85204.1, BAD85210.1, BAD85521.1
GCA_000009965.1	LAMASSU	LmuB | ------	BAD85206.1, BAD86400.1
GCA_000009965.1	RM	restriction | type II	BAD85347.1, BAD85649.1, BAD86419.1
GCA_000009965.1	RM	M_subunit | type I	BAD86030.1
GCA_000009965.1	RM	methyltransferase | type II	BAD84547.1, BAD85347.1, BAD85649.1, BAD86122.1, BAD86419.1
GCA_000009965.1	SEPTU	PtuA | ------	BAD84208.1

GCA_000009965.1	TA	AT | type II	BAD84206.1, BAD84253.1, BAD84254.1, BAD84316.1, BAD84327.1, BAD84366.1, BAD84381.1, BAD84402.1, BAD84410.1, BAD84524.1, BAD84529.1, BAD84533.1, BAD84561.1, BAD84562.1, BAD84732.1, BAD84884.1, BAD84922.1, BAD84981.1, BAD85077.1, BAD85098.1, BAD85106.1, BAD85152.1, BAD85162.1, BAD85186.1, BAD85202.1, BAD85217.1, BAD85257.1, BAD85261.1, BAD85265.1, BAD85316.1, BAD85373.1, BAD85444.1, BAD85480.1, BAD85528.1, BAD85664.1, BAD85700.1, BAD85834.1, BAD85899.1, BAD85941.1, BAD85951.1, BAD86004.1, BAD86185.1, BAD86195.1, BAD86257.1, BAD86343.1, BAD86418.1, BAD86450.1, BAD86494.1

GCA_000009965.1	TA	T | type II	BAD84205.1, BAD84252.1, BAD84255.1, BAD84317.1, BAD84326.1, BAD84365.1, BAD84380.1, BAD84401.1, BAD84411.1, BAD84523.1, BAD84530.1, BAD84532.1, BAD84560.1, BAD84563.1, BAD84645.1, BAD84731.1, BAD84863.1, BAD84885.1, BAD84923.1, BAD84980.1, BAD85078.1, BAD85099.1, BAD85107.1, BAD85151.1, BAD85154.1, BAD85161.1, BAD85187.1, BAD85203.1, BAD85216.1, BAD85258.1, BAD85262.1, BAD85264.1, BAD85317.1, BAD85374.1, BAD85445.1, BAD85479.1, BAD85665.1, BAD85699.1, BAD85833.1, BAD85898.1, BAD85940.1, BAD85952.1, BAD86005.1, BAD86184.1, BAD86194.1, BAD86256.1, BAD86344.1, BAD86417.1, BAD86451.1, BAD86495.1
GCA_000009965.1	WADJET	JetD | type III	BAD84987.1
GCA_000009965.1	WADJET	JetD | type I	BAD84987.1

GCA_000009965.1	WADJET	JetC | type III	BAD85206.1
GCA_000009965.1	ZORYA	ZorD | type I	BAD85336.1
